# FastCAT Accelerates Absolute Quantification of Proteins Using Multiple Short Nonpurified Chimeric Standards

**DOI:** 10.1021/acs.jproteome.2c00014

**Published:** 2022-05-13

**Authors:** Ignacy Rzagalinski, Aliona Bogdanova, Bharath Kumar Raghuraman, Eric R. Geertsma, Lena Hersemann, Tjalf Ziemssen, Andrej Shevchenko

**Affiliations:** † Max Planck Institute of Molecular Cell Biology and Genetics, 01307 Dresden, Germany; ‡ Center of Clinical Neuroscience, Department of Neurology, University Hospital Carl Gustav Carus, Technical University of Dresden, 01307 Dresden, Germany

**Keywords:** absolute quantification of proteins, MS Western, QconCAT, targeted quantitative proteomics, cerebrospinal fluid, neurodegeneration, neuroinflammation

## Abstract

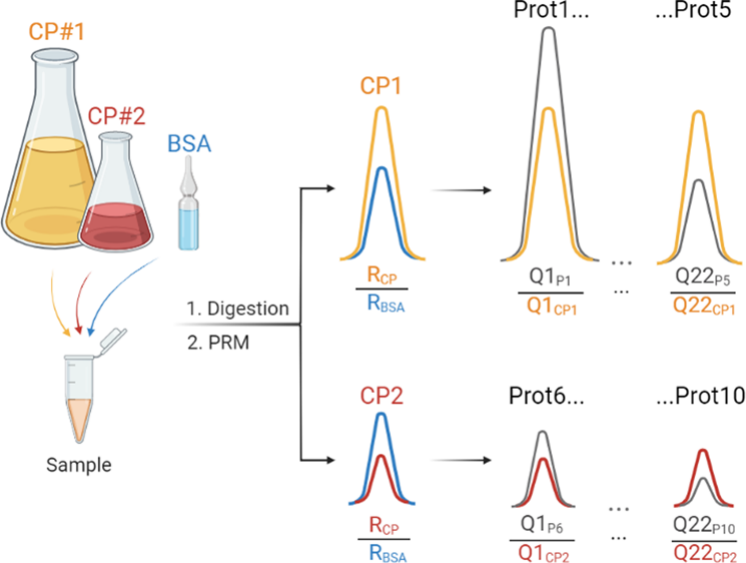

Absolute (molar) quantification of clinically relevant proteins determines their reference values in liquid and solid biopsies. The FastCAT (for Fast-track QconCAT) method employs multiple short (<50 kDa), stable-isotope labeled chimeric proteins (CPs) composed of concatenated quantotypic (Q)-peptides representing the quantified proteins. Each CP also comprises scrambled sequences of reference (R)-peptides that relate its abundance to a single protein standard (bovine serum albumin, BSA). FastCAT not only alleviates the need to purify CP or use sodium dodecyl sulfate-polyacrylamide gel electrophoresis (SDS-PAGE) but also improves the accuracy, precision, and dynamic range of the absolute quantification by grouping Q-peptides according to the expected abundance of the target proteins. We benchmarked FastCAT against the reference method of MS Western and tested it in the direct molar quantification of neurological markers in human cerebrospinal fluid at the low ng/mL level.

## Introduction

The role of absolute (molar) quantification of proteins is multifaceted. It determines stoichiometric ratios within molecular assemblies and metabolic pathways^1^ and relates them to the abundance of nonproteinous compounds, e.g., enzyme cofactors, lipids, or metabolites. It also provides reference values and ranges of their physiological variation for diagnostically important proteins in liquid and solid biopsies.^2^ Last but not least, it estimates the protein expression levels in cells and tissues serving as a quantitative denominator common to all *omics* sciences. In contrast to popular immunodetection methods (e.g., enzyme-linked immunosorbent assay (ELISA) or Western blotting),^3^ mass spectrometry quantifies proteins by comparing the abundance of endogenous quantotypic (Q)-peptides with corresponding synthetic peptide standards having the exactly known concentration. The protein concentration is then inferred from the concentrations of Q-peptides. However, protein and peptide properties are unique and may vary significantly.^4^ Furthermore, to support clinical diagnostics, it is often necessary to quantify a selection of disease-related proteins whose molar abundance differs by several orders of magnitude. It is therefore not surprising that, in contrast to the relative quantification, absolute quantification methods lack generality and unification.

Absolute quantification (reviewed in ref (5)) relies upon different types of internal standards, including (but not limited to) synthetic peptides (e.g., AQUA),^6^ full-length or partial protein sequences (e.g., PSAQ^7^ and QPrEST,^8^ respectively) and also chimeric proteins composed of concatenated quantotypic peptides from many different proteins (QconCAT)^9^ (reviewed in refs (10−13)). Powered by the recent advances in gene synthesis, QconCAT offers several appealing qualities such as the ease of multiplexing that enables targeted mid- to large-scale quantification of individual proteins, protein complexes, metabolic pathways^14^ or selections of clinically relevant proteins.^15,16^ QconCAT chimeras are expressed in *E. coli*, enriched, and purified by affinity chromatography, and their stock concentration is determined by amino acid analysis or some protein assays.^17^ Alternatively, an additional (secondary) peptide concatenated standard (PCS) could help to quantify multiple primary PCSs.^18^ Although cell-free expression systems^19,20^ improve the flexibility of QconCAT implementation, they do not alleviate the need to enrich, purify, and quantify the chimeric proteins (CPs). To simplify the quantification, the sequence of [Glu^1^]-Fibrinopeptide B could be included into the CP as a reference, although protein quantificaion based on a single synthetic peptide standard should be used with caution.^21^

By using GeLC-MS/MS, the MS Western workflow alleviated the need of making and standardizing a purified stock of CP. Also, CP standards were designed such that they included not only quantotypic (Q)-peptides but also several reference (R)-peptides.^22^ The bands of CP and of the reference protein (bovine serum albumin, BSA) were codigested with gel slabs containing target proteins, and the recovered peptides were analyzed by LC-MS/MS. Using R-peptides, the abundance of CP was referenced in situ to the exactly known amount of BSA, which is available as a NIST certified standard. Next, the abundance of target proteins was calculated from the abundance of CP assuming that its complete tryptic cleavage produced corresponding Q-peptides in an equimolar amount. MS Western quantification relied upon the concordant values (CV < 10%) obtained from multiple (usually 2 to 4) Q-peptides per protein of interest and took advantage of the high expression of CP in *E. coli*.^23,24^

Because of using sodium dodecyl sulfate (SDS) for proteins solubilization, GeLC-MS/MS could detect more membrane proteins. SDS-polyacrylamide gel electrophoresis (PAGE) also alleviated the need of purifying the CPs, yet it limited the analyses throughput. While assembling hundreds of Q-peptides into a large (up to 290 kDa) chimera is appealing, it is also inflexible because other proteins and/or peptides could not be added at will. Furthermore, the yielded Q-peptides are strictly equimolar, which hampers the quantification of proteins having drastic (more than 100-fold) differences in their abundance. This, however, is often required for the quantification of protein biomarkers.^25^

Here, we report on the FastCAT (for Fast-track QconCAT) method that preserves the accuracy and consistency of MS Western quantification, yet it is faster, more flexible, and easier to use particularly in translational proteomics applications. In contrast to MS Western, the FastCAT workflow relies on the parallel use of many relatively short (less than 50 kDa), nonpurified CPs that comprise Q-peptides for many target proteins but also scrambled R-peptides to reference each CP concentration to the same BSA standard.

## Experimental Section

### Chemicals and Reagents

LC-MS grade solvents (water, acetonitrile, and isopropanol), formic acid (FA), and trifluoroacetic acid (TFA) were purchased from Thermo Fisher Scientific (Waltham, MA) or Merck (Darmstadt, Germany). Trypsin and trypsin/Lys-C proteases (MS grade) were from Promega (Madison, WI); RapiGest detergent was from Waters (Eschborn, Germany), and other common chemicals were from Sigma-Aldrich (Munich, Germany). Polyacrylamide gradient gels (4–20%) were from Serva Electrophoresis GmbH (Heidelberg, Germany). Protein standards, glycogen phosphorylase (GP), alcohol dehydrogenase (ADH), enolase (ENO), and ubiquitin (UBI), were purchased as a lyophilized powder from Sigma-Aldrich. The reference protein standard (BSA, Pierce grade, in ampules) was from Thermo Fisher Scientific (Waltham, MA). Isotopically labeled amino acids (^13^C_6_,^15^N_4_-l-arginine (R) and ^13^C_6_-l-lysine (K)) were purchased from Silantes GmbH (Munich, Germany).

### Design and Expression of Chimeric Protein Standards

In total, six CP standards of different molecular weights (MWs) were designed and expressed. The general scheme of the CP design is presented in Figure S1, while other details including MWs, isotopic enrichment labeling efficiency, and full-length sequences are in Table S4. DNA sequences encoding CPs were codon-optimized for *E. coli* by the GenScript online tool and synthesized by GenScript (Piscathaway, NJ). CPs were produced using a pET backbone (Novagen) and *E. coli* BL21 (DE3) (*ΔargA ΔlysA*) strain auxotrophic for arginine and lysine supplemented with ^13^C_6_,^15^N_4_-l-arginine and ^13^C_6_-l-lysine as described.^22^ The *E. coli* strain^26^ was a kind gift from Professor Roland Hay (University of Dundee, UK).

### Sample Preparation for Proteomics Analyses

Polyacrylamide gels were stained with Coomassie CBB R250, and gel slabs corresponding to the targeted range of MW were excised. In-gel digestion with trypsin^22^ was carried out at the enzyme-to-substrate ratio of 1:50. *E. coli* proteins with 4 spiked standard proteins were in-pellet digested with trypsin (1:20) after proteins precipitation with isopropyl alcohol.^27^ Aliquots of cerebrospinal fluid (CSF) of 20 μL volume were in-solution digested with a trypsin/Lys-C protease mix (1:20) in the presence of RapiGest (Waters) detergent.^28^ CSF samples were obtained from patients diagnosed with multiple sclerosis and stored as freshly frozen aliquots. All patients gave their prior written consent. The study was approved by the institutional review board of the University Hospital Dresden (EK348092014).

### LC-MS/MS Analyses

LC-MS/MS was performed on a Q Exactive HF (Thermo Scientific, Germany) hybrid tandem mass spectrometer coupled with an Eksigent 400 nanoLC system (Sciex, Germany) using the Nanospray Flex ion source (Thermo Fisher Scientific, Germany). Protein digests were loaded onto a trap column for 5 min at 7 μL/min and separated on the Acclaim PepMap 100 column (C18, 3 μm, 75 μm × 150 mm) using 120 min gradients (5–45% B) at 300 nL/min in data-dependent acquisition (DDA) or parallel reaction monitoring (PRM) modes (as specified). DDA and PRM methods consisted of an MS1 scan from *m*/*z* 350 to 1700 with an automatic gain control (AGC) target value of 3 × 10^6^, maximum injection time (IT) of 60 ms, and targeted mass resolution (*R*_*m*/*z*=200_) of 60 000. The top-12 DDA method employed the precursor isolation window of 1.6 Th; AGC of 1 × 10^5^; maximum IT of 50 ms; *R*_*m*/*z*=200_ of 15 000; normalized collision energy (NCE) of 25%; dynamic exclusion of 30 s. The scheduled PRM method acquired MS/MS using 10 min retention time (RT) windows with the inclusion list of 99 precursors; precursor isolation window of 1.6 Th; AGC of 1 × 10^6^; a maximum IT of 80 ms; *R*_*m*/*z*=200_ of 30 000; NCE of 25%.

### Data Processing and Analysis

Raw LC-MS/MS data from DDA experiments were processed by Progenesis LC-MS v.4.1 (Nonlinear Dynamics, UK) software for RT alignment, peak picking, and extraction of the peptide features. To match peptides to target proteins, MS/MS spectra were searched by Mascot v.2.2.04 software (Matrix Science, London, UK) against a customized database containing sequences of all target proteins and the relevant (either *E. coli* or human) background proteome. The settings were as follows: precursor mass tolerance, 5 ppm; fragment mass tolerance, 0.03 Da; fixed modification, carbamidomethyl (C); variable modifications, acetyl (protein N-terminus) and oxidation (M); labels, ^13^C_6_ (K) and ^13^C_6_^15^N_4_ (R); cleavage specificity, trypsin with up to 2 missed cleavages allowed. All PRM data sets were analyzed with Skyline 21.1.0.278 software.^29^ Peak integration was inspected manually. Mass transitions in labeled and unlabeled peptides and matching retention time and peak boundaries confirmed the peptide identities. A minimum of five transitions was required for the correct identification of the targeted peptides. In addition, the comparison of the measured fragment spectrum to the in silico Prosit-derived^30^ library spectrum by the normalized spectral contrast angle that resulted in the library dot product (dotp) correlation values of 0.85 or higher was used.

## Results and Discussion

### Using Crude CP Standards for Protein Quantification

Trypsin cleavage of a purified CP produces Q- and R-peptides in a strictly equimolar concentration.^22,31,32^ However, in a crude extract of the expression host cells (*E. coli*), the balance between concentrations of individual Q-peptides could be affected by the nonproportional contribution of products of intracellular proteolysis of the CP and/or incomplete translation of its gene. Chimeric proteins are highly expressed in *E. coli* and are spiked into the analyzed sample in a minute (femtomole) amount.^22^ While CP purification reduces the concomitant load of *E. coli* proteins, it is unclear if their contribution to the overall compositional complexity is substantial.

**Figure 1:**
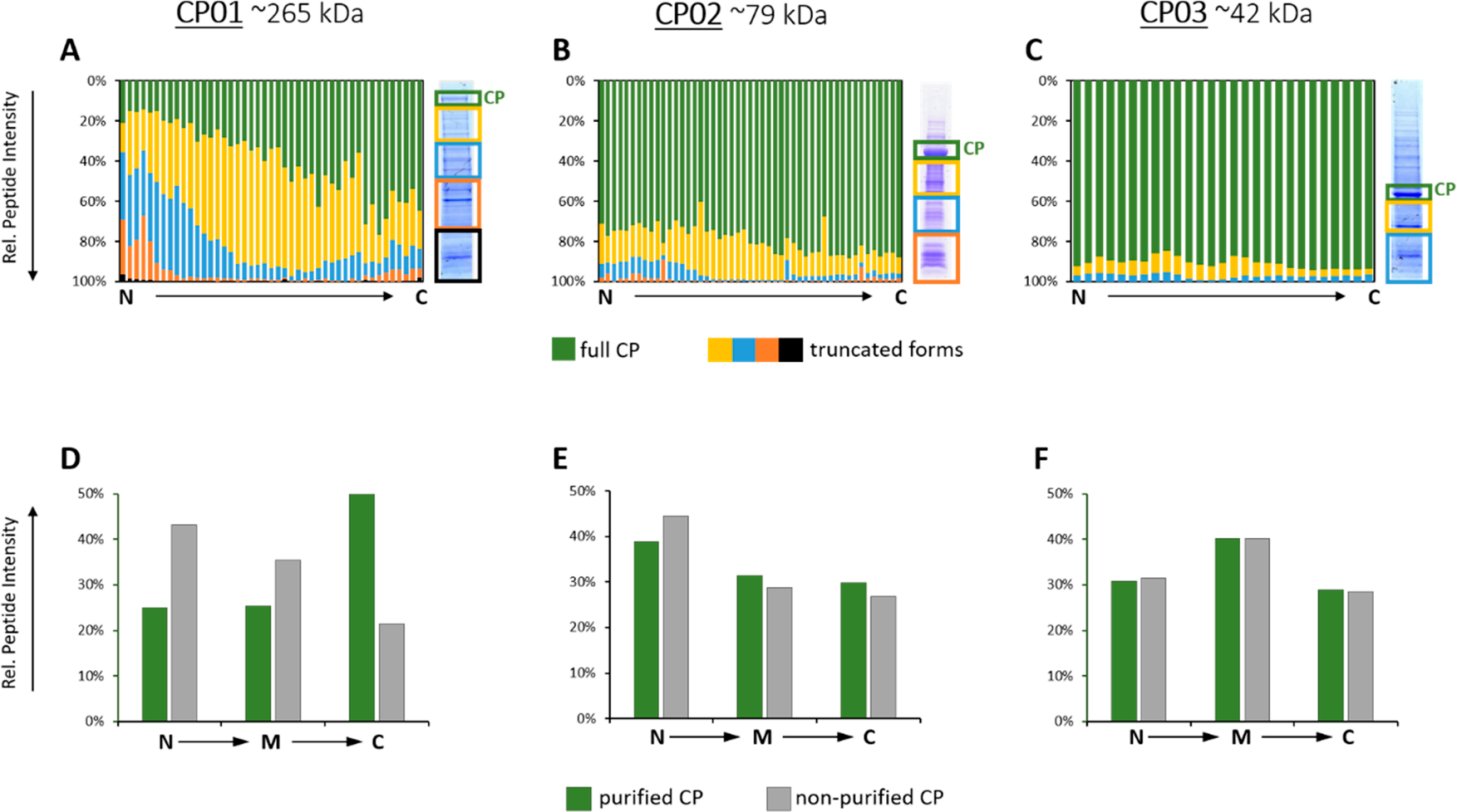
Truncation patterns for the chimeric proteins: CP01 ∼ 265 kDa (A, D), CP02 ∼ 79 kDa (B, E), and CP03 ∼ 42 kDa (C, F). The upper panels (A, B, C) present the relative abundance of peptides in SDS-PAGE slabs (*y*-axis) versus peptide positions in the CP sequence (*x*-axis); color-coding is exemplified at the right-hand side panel. Lower panels (D, E, F) present relative abundance of peptides from the purified CP (only CP band) versus the nonpurified CP (sum of all bands) for selected Q-peptides located at the N- and C-termini as well as in the middle of the CP sequence (“M”).

Therefore, we set out to test if full-length CP standards could be spiked directly as crude *E. coli* extracts with no prior purification. To this end, we selected three CP standards spanning a wide MW range (CP01 ∼ 265 kDa, CP02 ∼ 79 kDa, and CP03 ∼ 42 kDa) (Table S4). We loaded protein extracts onto 1D SDS-PAGE, excised the CP bands, and also sliced the entire gel slab in several MW ranges below the band of the CP and analyzed them separately by GeLC-MS/MS (Figure 1). We observed that the relative abundance of Q-peptides in each slice depended on the molecular weight of the full-length CP and its truncated forms, but also on the Q-peptide location within the CP sequence. In the 265 kDa CP, peptides located closer to its N-terminus were overrepresented and constituted 40% to 80% of total peptide abundances (Figure 1A). We also observed the same trend for the middle-size CP with a 20% to 40% fraction of truncated forms and a slight prevalence of N-terminally located peptides (Figure 1B). However, in the shortest (42 kDa) CP, the contribution of truncated forms was minor (ca. 10–15%) and independent of the peptide location (Figure 1C).

Because of the lower expression and higher fraction of proteoforms having incomplete sequences truncated elsewhere at the C-terminal side, larger CPs required extensive purification. In contrast, shorter (less than ca. 50 kDa) CPs were highly expressed, and cell lysate contained a much lower fraction of truncated forms with no location prevalence. Consistently, the relative abundances of Q-peptides at different locations within the CP sequence (N-terminal vs middle (“M”) vs C-terminal) in the purified CP and nonpurified CP (CP band together with truncation products) were very close (Figure 1E). Therefore, we concluded that spiking the total *E. coli* extract without isolating the full-length CP should not bias the quantification.

Since short CPs are highly expressed in *E. coli* such that they become the most abundant proteins in a whole lysate (Figure S2), the lysates are usually diluted more than 100-fold down to ca. 1 μM (or even lower) concentration of CP. Therefore, we wondered if adding the extract of *E. coli* containing an appropriate amount of nonpurified CPs still elevates the protein background. For reliable comparison, we mixed a volume of metabolically labeled extract containing typical working amounts of CP (ca. 100 fmol) with an equivalent volume of unlabeled extract containing ca. 500 ng of total protein. We then compared relative abundances of labeled and unlabeled forms of nine major *E. coli* proteins (Table S1) and observed that adding an extract with unpurified CP increased protein background by as little as 2% (Figure S3).

We therefore concluded that short ∼50 kDa CPs could be spiked into quantified samples as a total (crude) *E. coli* lysate with no prior purification. With that size, they would be encoding for 25 to 30 Q-peptides and 3 to 5 R-peptides within a typical CP construct.^22^

### FastCAT Workflow: The Concept and Its Validation

We reasoned that, by employing short nonpurified CPs, a targeted absolute quantification workflow (termed FastCAT for Fast-track QconCAT) could significantly accelerate the analysis. Besides Q-peptides used to quantify target proteins, a typical FastCAT construct contains multiple (usually 3 to 5) R-peptides used to determine the in situ CP concentration by referencing it to the known amount of spiked-in BSA. Hence, the FastCAT workflow requires neither CP purification (externally as a stock or using the band from gel electrophoresis) nor separate determination of its concentration, while the multipeptide quantification procedure is the same as in MS Western.

We cross-validated FastCAT by comparing it against MS Western.^22^ To this end, we prepared an approximately equimolar (ca. 1 μM) mixture of the 4 standard proteins (GP, UBI, ADH, and ENO) and spiked it (ca. 20 pmol of each protein) into the *E. coli* background (ca. 50 μg of total protein). We determined their exact quantities by MS Western using 42 kDa (CP03) as an internal standard.^22^ In parallel, we quantified them by the FastCAT protocol using the same 42 kDa construct but without 1D SDS-PAGE. Importantly, we checked the digestion completeness for both methods by comparing relative abundances of labeled Q-peptides in CP03 and corresponding unlabeled peptides in the endogenous proteins. The difference in protein quantities determined by FastCAT and MS Western was below 15% for all proteins (Table 1).

**Table 1:** Comparison of Protein Quantitation by FastCAT and MS Western

	calculated amount^a^ [fmols/column]		
protein	MS Western	FastCAT	quantification error [%]
GP	197.5	189.9	3.8
UBI	nq	231.2	nq
ADH	205.9	183.0	11.1
ENO	222.9	208.9	6.3

a Proteins were quantified by averaging the amounts calculated using 3 to 5 Q-peptides. UBI was not detected/quantified (nq) by MS Western.

**Figure 2:**
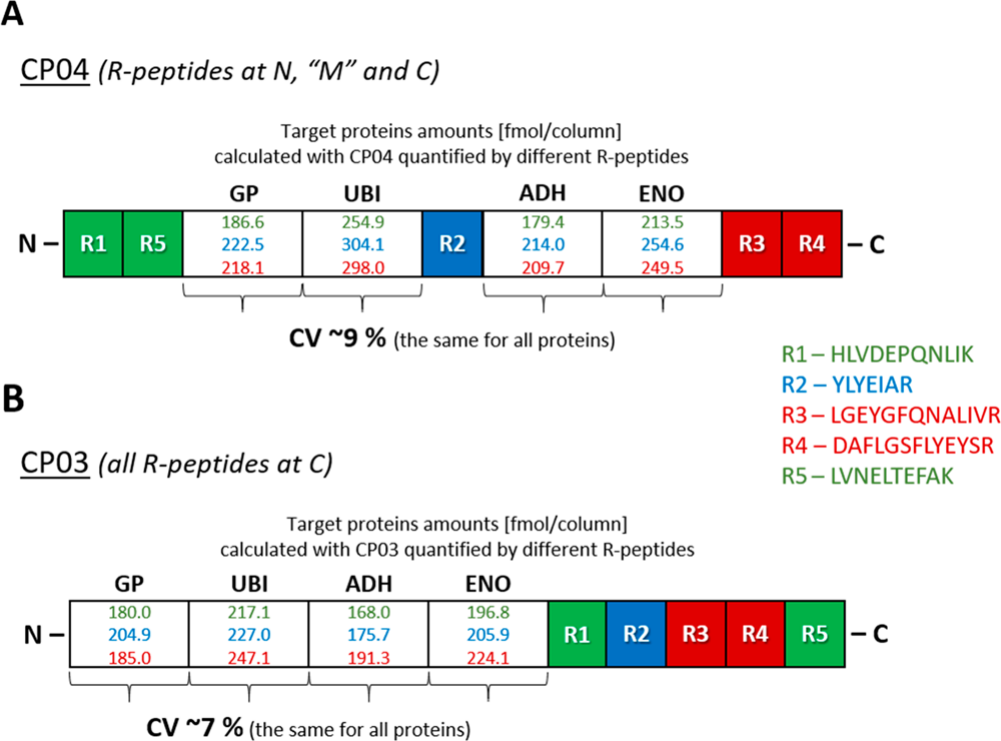
Impact of the location of R-peptides within the CP sequence on the protein quantification. Comparison of the target protein quantification using CP04 and N- vs “M” vs C-terminus R-peptides as well as using CP03 and the same groups of peptides but all positioned at the C-terminus.

Since FastCAT workflow implies no CP purification, we additionally checked if the location of Q- and R-peptides within the CP backbone affected the quantification. To this end, we designed another CP standard, CP04, having the same Q-peptides as in CP03, but in which the same five R-peptides (R1–R5) were distributed over the entire CP sequence, in contrast to a single block of R-peptides at the C-terminus of CP03 (see Figure 2).

This experiment resulted in two major findings. First, using CP03 and CP04 independently led to the concordant quantification. With all R-peptides used for the calculation of the CP abundances, the quantities of target proteins differed by less than 15%. Second, the quantification was practically unaffected by the location of the R-peptides. Indeed, using differently positioned R-peptides (N (R1/R5) vs “M” (R2) vs C (R3/R4)) in CP04 led to the quantification of all target proteins with a CV of ca. 9% (Figure 2A). This relatively minor variability could not be solely attributed to R-peptide placement. We note that the CV of ca. 7% was observed when the target proteins were quantified using CP03 with R-peptides (R1/R5 vs R2 vs R3/R4) placed at the C-terminus, as was for CP04 (Figure 2B).

We therefore concluded that in the FastCAT workflow the CP design including the location of R-peptides in its sequence have no major impact on the protein quantification.

### Multiplexing of FastCAT

Relatively short CP standards having MW of 40 to 50 kDa will typically comprise 20 to 30 Q-peptides. Since the robust protein quantification typically requires 3 to 5 peptides per protein, one CP should enable the quantification of 5 to 8 individual proteins of, preferably, similar abundance. Hence, there is a clear need to multiplex the FastCAT quantification capacity.

**Figure 3:**
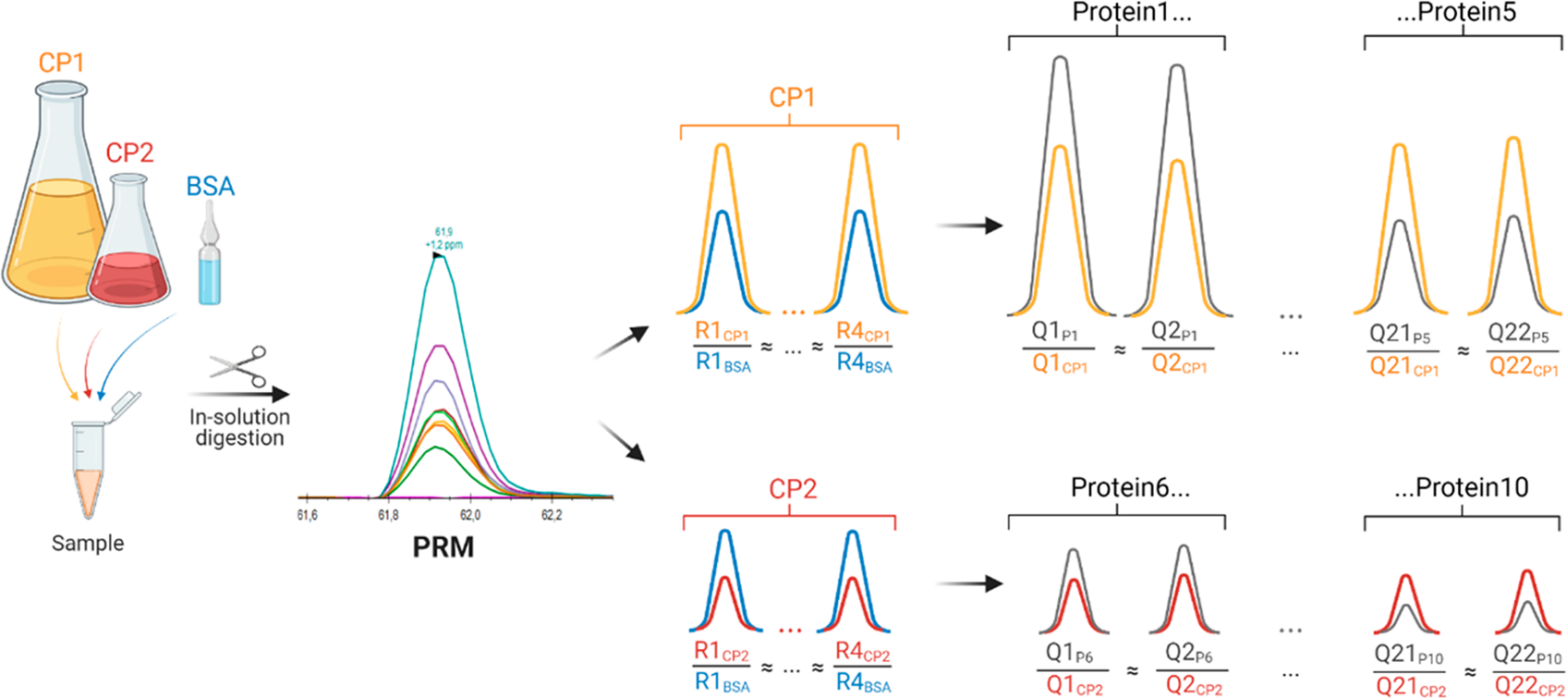
FastCAT workflow for absolute quantification of proteins. Target proteins (Protein1 to Protein10) are quantified using multiple (here, two) unpurified metabolically labeled chimeric protein standards CP1 and CP2. As an example, here CP1 contains two Q-peptides for each of the five proteins P1 to P5; CP2 – for the proteins P6 to P10, together with four R-peptides having native or scrambled sequences from BSA. The amount of spiked CP1 and CP2 is adjusted to the expected range of target protein concentrations; the concentration of BSA is known. Proteins are digested with trypsin and analyzed by PRM LC-MS/MS. First, CP1 and CP2 are quantified by comparing peaks of their “heavy” reference peptides (R1_CP1_ to R4_CP1_; R1_CP2_ to R4_CP2_) and matching (R1_BSA_ to R4_BSA_) peptides from BSA. Then, the concentration of each target protein (e.g., P1) is calculated from peak areas of endogenous Q-peptides (Q1_P1_ and Q2_P1_) and of matching “heavy” Q-peptides (Q1_CP1_ and Q2_CP1_) whose concentration equals CP1. Other proteins, including those covered by CP2, are quantified similarly..

We therefore propose to group proteotypic peptides into CPs according to the expected abundance of the target proteins and then to use multiple CPs in parallel to eventually cover the desired number of proteins (Figure 3). However, the abundance of different CPs should be referenced to the same spiked-in BSA standard. We achieved it by including reference peptides with the scrambled sequences (R_s_-peptides)^33^ that, nevertheless, elicit a very similar response in MS1 spectra compared to the corresponding native R_n_-peptides. For better sensitivity, the targeted analysis would also require PRM for the quantification of CPs in the same LC-MS/MS run. Therefore, for each R_n_/R_s_-peptide pair, we selected a representative combination of fragment ions that adequately reflected the peptide abundance^34,35^ and, hence, enabled parallel quantification of multiple CPs.

**Figure 4:**
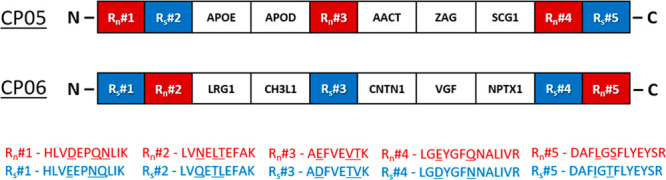
Scheme of CP05 and CP06 constructs comprising the native (R_n_#1–R_n_#5) and scrambled (R_s_#1–R_s_#5) forms of 5 BSA peptides (shown as one block/peptide) as well as the multiple Q-peptides from 10 target proteins (shown as one block/protein).

To this end, we designed CP05 and CP06 proteins (see Figure S4 for amino acid sequences and Figure S5 for the distribution of their truncated forms) containing 42 Q-peptides from 10 selected human proteins (these CPs were further used in the case study described in the next section). They also comprised 10 R-peptides as 5 pairs of native (R_n_) and scrambled (R_s_) sequences. However, R-peptides were placed into CP05 and CP06 such that they contained 3 native plus 2 scrambled R-peptides and 2 native plus 3 scrambled R-peptides, respectively (Figure 4). To emulate the impact of the protein background, we spiked these CPs in ca. 1 μM concentration into a 20 μL aliquot of human cerebrospinal fluid (CSF) and analyzed their tryptic digests by the method of PRM.

**Figure 5:**
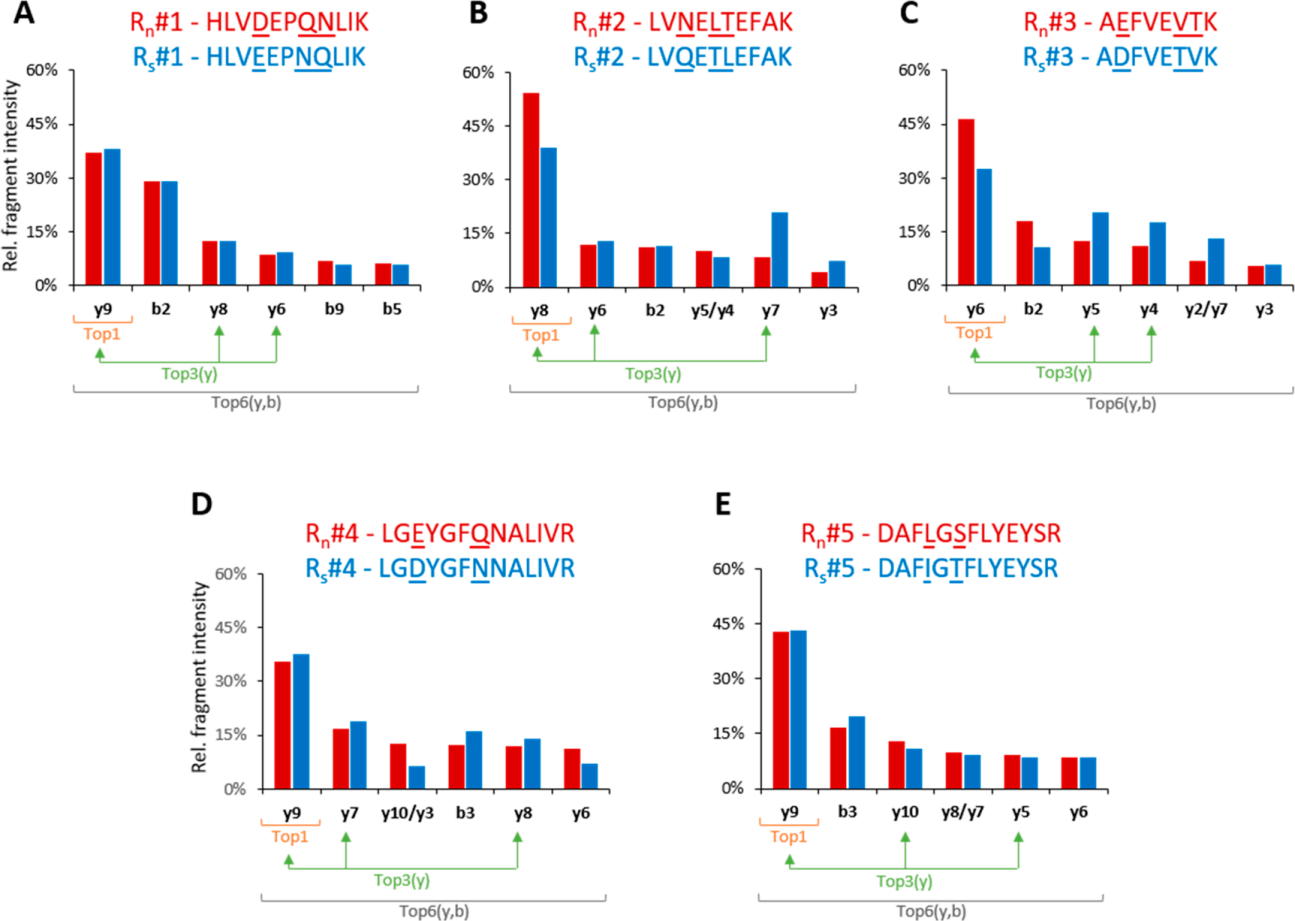
Relative intensties of the six most abundant *y-* and *b-* fragment ions from the native (BSA/light, red bars) and scrambled (CP/heavy, blue bars) R-peptides. In four out of five pairs, one fragment ion within the top 6 did not match between the native and scrambled forms. The nonmatching fragments were presented together to contrast the fragmentation differences.

For each R_n_/R_s_-peptide pair, we compared the distribution of the intensties of the most abundant fragments within *y*- and *b*-ions (see Figure S6 for the corresponding MS2 spectra). Out of five pairs, three pairs produced very similar profiles, while the two other pairs mismatched (Figure 5).

**Figure 6:**
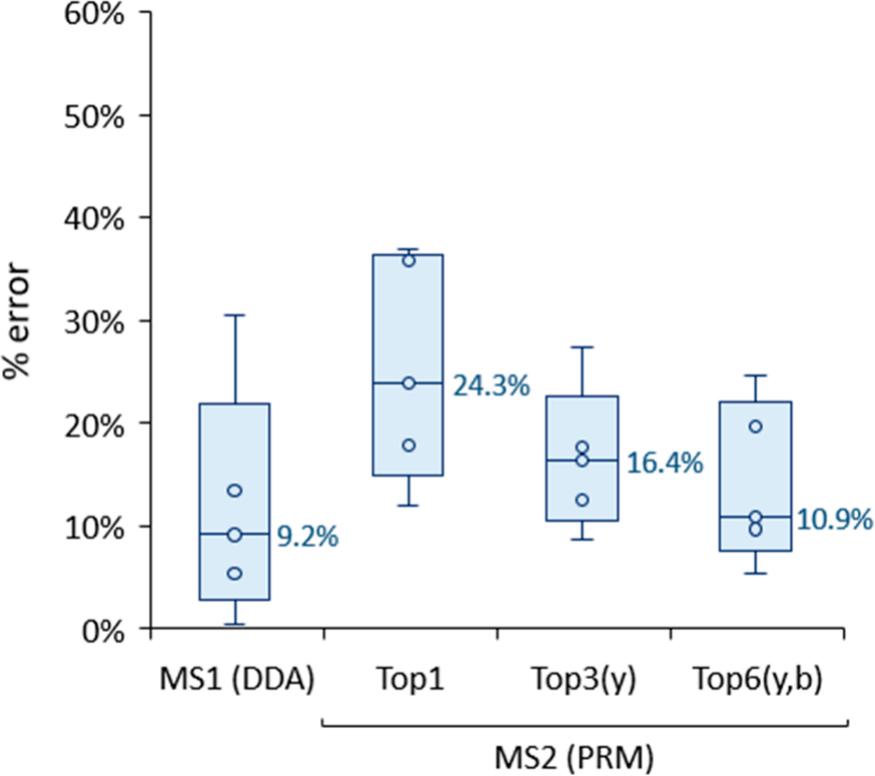
Relative error in the quantification of CPs using R_s_-peptides and either MS1 or MS2 (summed) intensities of different fragment ions. Top1 (the top fragment ion); Top3(*y*) (sum of the top three *y-*ions, matching between R_n_ and R_s_); Top6(*y,b*) (sum of the top six fragments within *y-* and *b-*ions). Each box contains information from 5 R_s_-peptides (detailed information is provided in Table S2), while the median values are given next to the boxes. Each box plot displays the median (line), the 25th and 75th percentiles (box), and the 5th and 95th percentiles (whiskers).

Next, we assessed the concordance of the in situ PRM-based quantification of CPs using each R_s_-peptide and R_n_-peptide. To do this, we considered three PRM quantification scenarios on the basis of the selection of different combinations of fragment ions—Top1, Top3(*y*), and Top6(*y,b*) (as exemplified in Figure 5), and compared them to the values computed from the intensities of MS1 peaks (see Table S2). As expected, the Top1 approach not only resulted in the highest error (>35%) for the two mismatching R_n_/R_s_ (#2 and #3) pairs, but also revealed the highest overall (for all R_n_/R_s_ pairs) discordance with MS1 (Figure 6). In contrast, summing up the abundances of more fragments in Top3(*y*) or Top6(*y,b*) scenarios compensated minor differences in fragmentation patterns of R_n_/R_s_-peptides. We note that, for selected reaction monitoring (SRM) experiments performed on low mass resolution instruments that cannot follow many transitions in parallel and where using *b*-ions should be avoided,^36^ the Top3(*y*) approach could be the most practical compromise.

However, for PRM-based quantification, the sum of intensities of the 6 most abundant *y-* and *b*-fragment ions will lead to the most consistent estimates with lower than 20% difference of MS1-based determinations (Figure 6). Eventually, within the two CPs, the relative abundances of all used (both native and scrambled) R-peptides measured via Top6(*y,b*) fragments differed by less than 5% (Figure S7).

We therefore concluded that FastCAT quantification can be multiplexed by simultaneously using several CPs comprising multiple scrambled peptides of BSA. Quantities of individual CPs could be referenced to the same BSA standard also by the PRM analysis that relies on the summation of intensitites of six most abundant *y-* and *b-*ions for each R_s_ (in the CP) and R_n_ (in BSA standard) peptide pair.

### Case Study: Absolute Quantification of Neurological Protein Markers in the Human CSF

Multiple sclerosis is an immune-mediated demyelinating and neurodegenerative disease of the central nervous system (CNS), which is accompanied by blood–brain barrier disruption, infiltration of immune cells into the CNS, nerve fiber demyelination, and axonal loss.^37^ It alters the CSF proteome, and monitoring the levels of protein markers by common clinical chemistry methods (e.g., ELISA) aids in its molecular diagnostics.^38^ However, these methods suffer from low concordance, limited scope, and substantial costs. Here, we employed FastCAT to determine the molar concentration of a selection of protein markers having a broad range of physicochemical properties, molar abundance, and magnitude of response toward the disease.

We obtained 11 samples of CSF from five (four female and one male), 30 to 61 year old patients that were diagnosed with relapsing-remitting multiple sclerosis. CSF was drawn from each patient at two time points: the first puncture was performed during the initial diagnostics, while the second (and, for one patient, also the third) puncture was performed ca. 2 years later prior to a planned treatment switch to validate that no significant inflammation and neurodestruction occurred. On the basis of clinical indications, ten protein markers were selected out of ca. 700 proteins detected in a pooled CSF sample by the preliminary experiment. Those included two major lipoproteins (APOE and APOD); inflammation-related glycoproteins (AACT, ZAG, and LRG1); markers of axonal (CNTN1) and synaptic (NPTX1 and VGF) related disorders; a member of the granins family (SCG1); a neuroinflammatory marker (CH3L1) typically increased in patients with multiple sclerosis.^39,40^ We then selected 42 Q-peptides and assembled them in CP05 and CP06 (both mentioned above) according to the arbitrary abundance of target proteins.

The method precision was evaluated by processing and analyzing the pooled CSF sample in triplicate (Figure S7). For both CP and target protein peptide and protein levels, the median coefficient of variation was below 6%. Importantly, peptides originating from the same protein led to their highly concordant quantifications as exemplified by a median CV of ca. 12%.

The median values and the ranges of variation of the target proteins concentration are reported in Table 2, which also includes Q-peptides and the estimates of concordance for the independent quantification by multiple peptides. Concentration ranges determined by FastCAT corroborated previously reported SRM and PRM determinations. For instance, similar concentration ranges were obtained for APOE,^41,42^ AACT,^43^ SCG1,^44^ and CH3L1.^45^ At the same time, the concordance with ELISA measurements was limited for both FastCAT and published SRM/PRM values. While ranges determined by ELISA for AACT,^46^ LRG1,^47,48^ CH3L1,^45,49,50^ and CNTN1^46^ were close to those obtained by FastCAT, the levels for both apolipoproteins were discordant (yet, again, concordant with SRM/PRM).^51−54^ This, however, is consistent with the known discrepancy between ELISA and mass spectrometry measurements.^41^ The molar concentration of ZAG, VGF, and NPTX1 was not reported previously.

**Table 2:** Marker Proteins Concentrations (Ranges and Median) Determined in 11 CSF Samples from 5 Patients with Multiple Sclerosis as well as UniProt Accession Numbers, Q-Peptides Used for Quantification, Intraprotein Concordance (Median CV from All Samples), and Previously Reported Concentrations Determined by Mass Spectrometry and/or ELISA^a^

protein	UniProt accession	Q-peptides	intraprotein peptide concordance (median CV [%])	concentration range (median) [ng/mL]	reported concentration range (method and ref) [ng/mL]
APOE	P02649	SELEEQLTPVAEETR; LGPLVEQGR; QWAGLVEK; LAVYQAGAR	7.5	922.1–2075.6 (1807.8)	900–3000 (SRM);^41^ 2964–3112 (PRM);^42^ 7000–12 000 (ELISA);^53^ 4000–13 000 (ELISA)^52^
APOD	P05090	NILTSNNIDVK; NPNLPPETVDSLK; VLNQELR; WYEIEK	5.7	1060.4–1327.3 (1256.6)	3000–12 000 (ELISA)^54^
AACT	P01011	ITLLSALVETR; NLAVSQVVHK; AVLDVFEEGTEASAATAVK; ADLSGITGAR	10.1	540.9–1127.3 (724.0)	126–2954 (PRM);^43^ 1400–3900 (ELISA)^46^
ZAG	P25311	EIPAWVPFDPAAQITK; WEAEPVYVQR	9.0	85.2–113.2 (96.3)	n/a
SCG1	P05060	NYPSLELDK; NYLNYGEEGAPGK; WQQQGDLQDTK	12.4	388.6–942.3 (850.1)	300–900 (SRM)^44^
LRG1	P02750	VAAGAFQGLR; GQTLLAVAK	14.0	27.1–114.3 (68.0)	25–350 (ELISA);^47^ 90–800 (ELISA)^48^
CH3L1	P36222	FPLTNAIK; ILGQQVPYATK; VTIDSSYDIAK; GNQWVGYDDQESVK	5.8	25.7–53.8 (35.2)	35–254 (SRM);^45^ 70–160 (ELISA);^45^ 29–182 (ELISA);^49^ 30–350 (ELISA);^50^
CNTN1	Q12860	FIPLIPIPER; ASPFPVYK	12.4	148.3–268.6 (238.9)	20–300 (ELISA)^46^
VGF	O15240	NSEPQDEGELFQGVDPR; THLGEALAPLSK	30.9	60.6–175.9 (111.0)	n/a
NPTX1	Q15818	FQLTFPLR; TPAAETLSQLGQTLQSLK	5.6	37.2–86.0 (72.0)	n/a

a Proteins: APOE (apolipoprotein E), APOD (apolipoprotein D), AACT (alpha-1-antichymotrypsin), ZAG (zinc-alpha-2-glycoprotein), SCG1 (secretogranin-1/chromogranin B), LRG1 (leucine-rich alpha-2-glycoprotein 1), CH3L1 (chitinase-3-like protein), CNTN1 (contactin-1), VGF (neurosecretory protein VGF), and NPTX1 (neuronal pentraxin-1).

Concentrations determined in individual patients (Table S3) were stable over the two year period of treatment and, except in one patient, clustered together at the PCA plot (Figure S9). The PCA plot singled out one female patient presumably because of her older age although protein concentrations in both of her biopsies were concordant.

Several trends (e.g., increase in APOD and ZAG; decrease in NPTX1) corroborated previous reports.^55−57^ At the same time, there was no consistent change in the levels of prospective multiple sclerosis markers SCG1 and LRG1.^48,58^

The amount of spiked CP05 standard was ca. 10-fold higher than that of CP06 (309 fmol/column vs 33 fmol/column, respectively). For better consistency, BSA was spiked at some intermediate amount (100 fmol/column). In this way, PRM covered a 100-fold range of concentrations from ca. 20 ng/mL for CH3L1 to ca. 2000 ng/mL for APOD without compomising interpeptide quantification consistency. Considering the signal-to-noise ratios, PRM was not even close to the limit of detection and, in principle, should allow us to reach 10-fold higher sensitivity if the appropriate amount of yet another CP standard is spiked into CSF samples. Taken together, we demonstrated that FastCAT supported direct molar quantification of 10 neurological protein markers in CSF at low ng/mL levels and delivered better than a 100-fold dynamic range and good (CV < 20%) interpeptide quantification consistency based on two to four peptides per each protein.

## Conclusion and Perspectives

The absolute quantification offers heavily missing data on the molar concentrations (or molar abundances) of proteins in cells, tissues, and biofluids. The FastCAT workflow takes advantage of the flexibility and ease of use of peptide-based quantification. It relies on a large number of peptide standards having the exactly known and equimolar concentration that are produced in situ and quantified in the same LC-MS/MS run together with peptides from target proteins.

In this work, we used two CPs to quantify 10 neurological markers in the human CSF. However, FastCAT offers ample capabilities for multiplexing. First, re-engineering reference peptides combined with alternative metabolic labeling (e.g., using different combinations of commercially available isotopologues of arginine and lysine) could produce dozens of R-peptides to accommodate many more unique CPs. A rough calculation suggests that we might only need to design five more R-peptide variants to employ 10 CPs in parallel. Assuming each 50 kDa CP comprises 3 Q-peptides per target protein, this would already cover 100 proteins in a single LC-MS/MS run. Modern analytical solutions such as SureQuant or PRM-Live offer intelligent real-time PRM scheduling and enable the quantification of hundreds of peptide precursors without compromising the sensitivity and throughput. Furthermore, PRM quantification could be organized in a “modular” fashion by combining CPs of the desired Q-peptides composition and having similar concentration ranges. CP standards are produced by expressing synthetic genes in *E. coli* and, because of consistently high expression levels (Figure S2), could be used directly from a host cell lysate without their prior purification.

In the future, it might be practical to set up a publicly available repository of plasmids encoding CPs. This will improve the analyses consistency and, eventually, bring the absolute quantification availability and performance closer to clinical chemistry requirements. While the throughput of LC-MS/MS quantification could hardly compete with ELISA, its accuracy, independence of antibodies, quantification transparency, and analytical flexibility, including the compatibility with major protocols for biochemical enrichment and robotic sample preparation, might be appealing for translational applications. We also argue that the interested laboratories should work together toward benchmarking and validating the absolute quantification methods by ring trials that are now common in neighboring omics fields, e.g., lipidomics.^59^

## Supporting Information Available

Figure S1, scheme of chimeric protein (CP); Figure S2, gel images of short CPs; Figure S3, *E. coli* background in nonpurified CP; Figure S4, sequences of CP05/CP06; Figure S5, truncation patterns for CP05/CP06; Figure S6, tandem mass spectra for R_n_/R_s_-peptides; Figure S7, relative R_n_/R_s_-peptide abundances for CP05/CP06 vs BSA; Figure S8, precision of the FastCAT method; Figure S9, PCA plot for CSF proteins from patients; Table S1, *E. coli* background proteins; Table S2, error in quantification of CPs by R_s_-peptides; Table S3, protein concentrations in CSF; Table S4, sequences for all CPs (PDF)

## Notes

Raw DDA data have been deposited at the ProteomeXchange Consortium via the PRIDE partner repository with the data set identifier PXD030728. PRM data processed by Skyline are available at Panorama Public at https://panoramaweb.org/FastCAT.url.
