# Supplementary material for: FastCAT Accelerates Absolute Quantification of Proteins Using Multiple Short Nonpurified Chimeric Standards
Source: J Proteome Res. 2022 May 13;21(6):1408–17. doi: 10.1021/acs.jproteome.2c00014 (PMC9171895; doi:10.1021/acs.jproteome.2c00014)
Supplement: Supplementary file 1 — pr2c00014_si_001.pdf [file pr2c00014_si_001.pdf]

Supporting Information

FastCAT accelerates absolute quantification of proteins by using multiple short non-purified chimeric standards

Ignacy Rzagalinski[1], Aliona Bogdanova[1], Bharath Kumar Raghuraman[1], Eric R. Geertsma[1], Lena Hersemann[1], Tjalf Ziemssen[2], Andrej Shevchenko[1]*

[1]*Max Planck Institute of Molecular Cell Biology and Genetics, Dresden, Germany*
[2]*Center of Clinical Neuroscience, Department of Neurology, University Hospital Carl Gustav Carus Dresden, Technical University of Dresden, Dresden, German*

<u>Table of Content</u>

Figure S1	Scheme of chimeric protein (CP)
Figure S2	SDS-PAGE gel images of short CPs
Figure S3	*E. coli* background in non-purified CP
Figure S4	Amino acid sequences of CP05/CP06
Figure S5	Truncation patterns for CP05/CP06
Figure S6	Tandem mass spectra for R_n/R_s-peptides
Figure S7	Relative R_n/R_s-peptides abundances for CP05/CP06 vs. BSA
Figure S8	Precision of FastCAT method
Figure S9	PCA plot for CSF proteins from patients with multiple sclerosis
Table S1	*E. coli* background proteins used for comparison in Figure S2
Table S2	Error in quantification of CPs by R_s-peptides
Table S3	Protein concentrations in CSF from individual patients
Table S4	Amino acid sequences for all used CPs

Chimeric protein (CP) standard

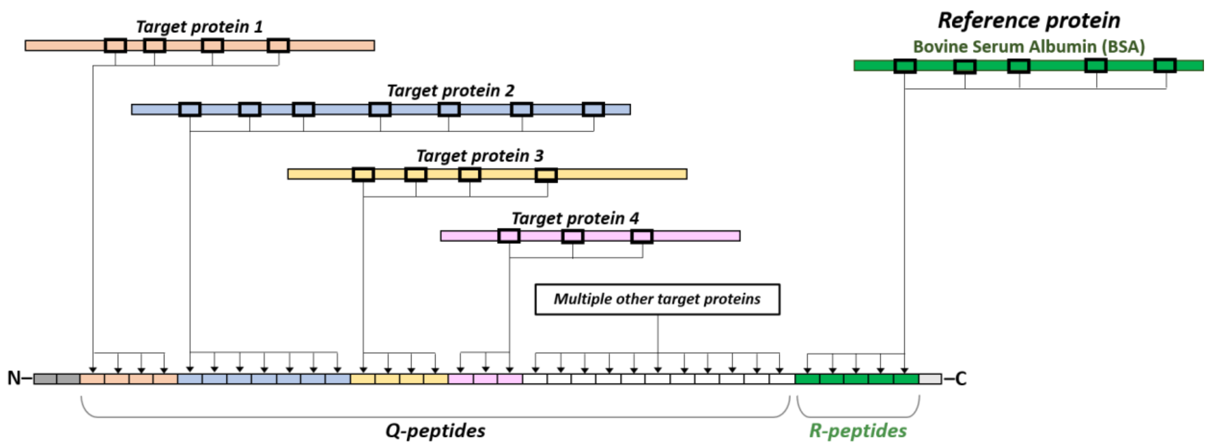

Figure S1. Scheme of chimeric protein (CP) standards used in this work. A typical CP comprises quantotypic Q-peptides selected from the sequences of target proteins and reference R-peptides selected from bovine serum albumin (BSA). They were concatenated *in-silico* into a chimeric sequence, whose N- and C-termini contain additional tag sequences (in gray) for R- and Q- peptides protection and (optionally) for the affinity purification.

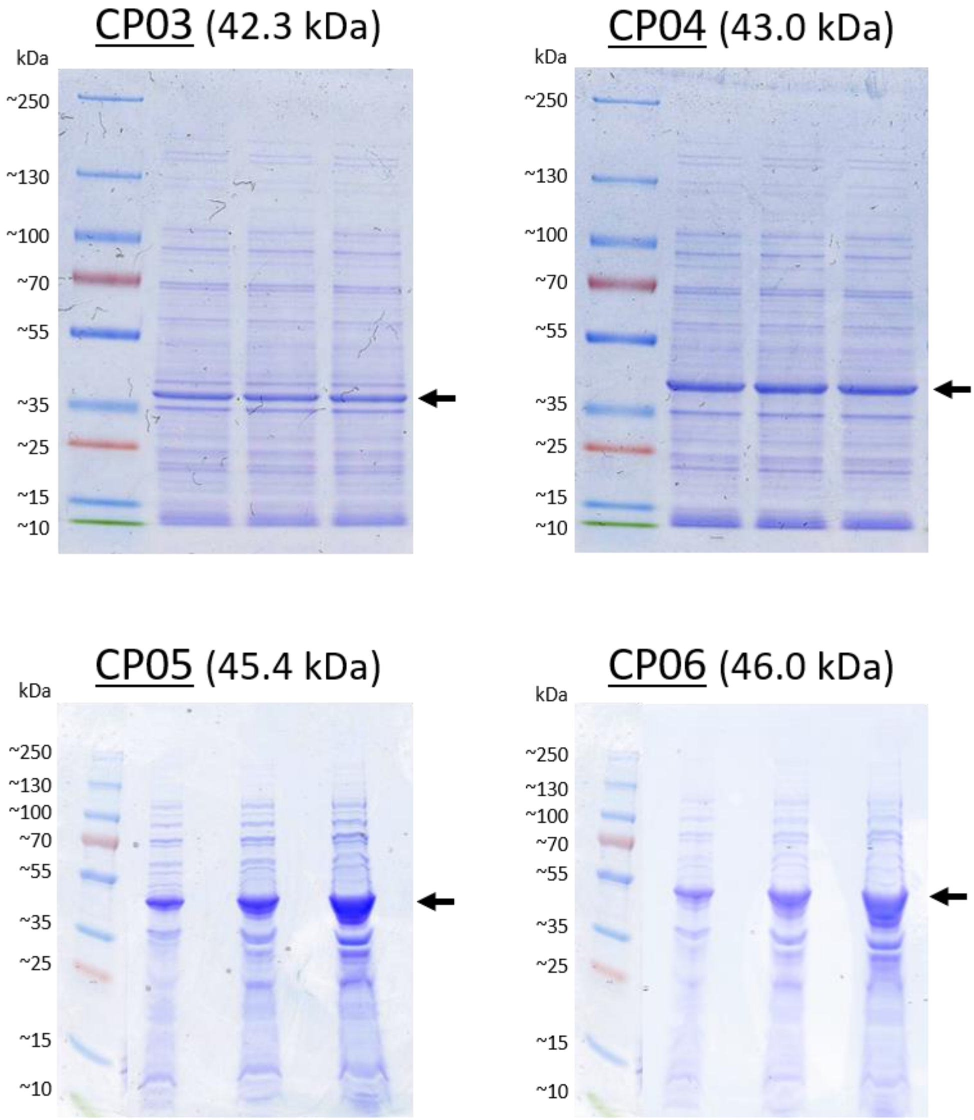

Figure S2. Images of Coomassie stained SDS-PAGE gels of four short (<50 kDa) CPs used in this study. Gel bands corresponding to the full-length CPs are designated with arrows.

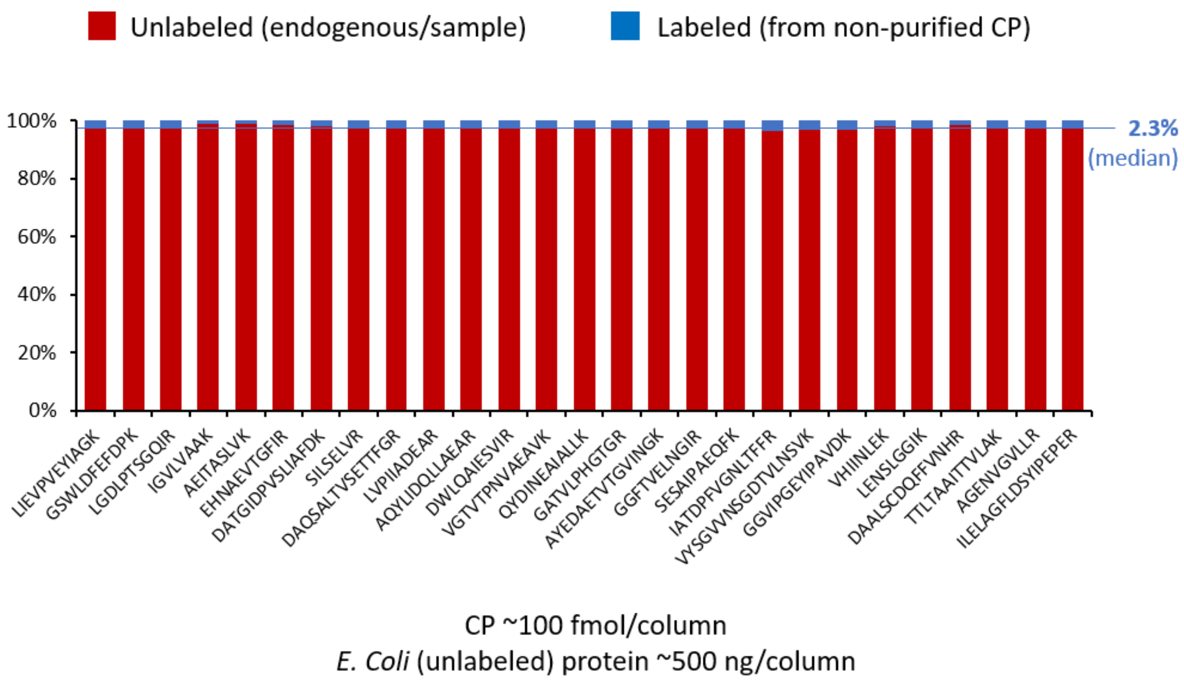

Figure S3. Contribution of extra background from the expression host (*E. coli*) introduced by spiking a unpurified CP standard (labeled, blue bars) compared to unlabeled *E. coli* proteins (unlabeled/endogenous, red bars). The protein amounts are typical for FastCAT experiments with CP at approximately 100 fmol and total protein of 500 ng loaded on column. The contribution of background (labeled) is ~2 % of the total protein. Nine abundant *E. coli* proteins (mainly ribosomal proteins and elongation factors) were considered. Their quantification was based on the abundance of at least three peptides having consistent L/H ratios (see Table S1). These peptides were detected as predominantly doubly charged ions, did not have missed cleavage sites and cysteine and methionine residues. Moreover, CP labeling efficiency exceeded 99 % and therefore all unlabeled peptides originated from background *E. coli* proteins.

A CP05

MGSAWSHPQFEKGGGSGGGSGGSAWSHPQFEKLEVLFQGPAAAKVFADYEEYVKDFYELEPHKV
AAAFPGDVDRGLAGVENVTELKHLVDEPQNLIKLVQETLEFAKSELEEQLTPVAEETRLGPLVEQGR
AATVGSLAGQPLQERQWAGLVEKLAVYQAGARNILTSNNIDVKIPTTFENGRNPNLPPETVDSLKV
LNQELRWYEIEKAEFVEVTKITLLSALVETRNLAVSQVVHKAVLDVFEEGTEASAATAVKADLSGITG
ARHVEDVPAFQALGSLNDLQFFRYSLTYIYTGLSKEIPAWVPFDPAAQITKQKWEAEPVYVQRNYPS
LELDKNYLNYGEEGAPGKGEAGAPGEEDIQGPTKWQQQGDLQDTKLGEYGFQNALIVRDAFIGTF
LYEYSRGSGHHHHHH

Apolipoprotein E (APOE) – Apolipoprotein D (APOD) – Alpha-1-antichymotrypsin (AACT) – Zinc-alpha-2-glycoprotein (ZAG) – Secretogranin-1 (SCG1)
BSA (R-)peptides: native and scrambled

B CP06

MGSAWSHPQFEKGGGSGGGSGGSAWSHPQFEKLEVLFQGPAAAKVFADYEEYVKDFYELEPHKV
AAAFPGDVDRGLAGVENVTELKHLVEEPNQLIKLVNELTEFAKVAAGAFQGLRGQTLLAVAKTLDLG
ENQLETLPPDLLRENQLEVLEVSWLHGLKFPLTNAIKILGQQVPYATKTLLSVGGWNFGSQRVTIDS
SYDIAKGNQWVGYDDQESVKADFVETVKFIPLIPIPERASPFPVYKTDGAAPNVAPSDVGGGGGR
VQVTSQEYSARNSEPQDEGELFQGVDPRTHLGEALAPLSKLADLASDLLLQYLLQGGARVGEEDEE
AAEAEAEAEEAERFQLTFPLRTPAAETLSQLGQTLQSLKALSGNVIAWAESHIEIYGGATKLGDYGFN
NALIVRDAFLGSFLYEYSRGSGHHHHHH

Leucine-rich alpha-2-glycoprotein (A2GL) – Chitinase-3-like protein (CH3L1) – Contactin-1 (CNTN1) – Neurosecretory protein VGF (VGF) – Neuronal pentraxin-1 (NPTX1)
BSA (R-)peptides: native and scrambled

Figure S4. Amino acid sequences of the CP05 and CP06 constructs used for the quantification of 10 neurological protein markers in CSF samples.

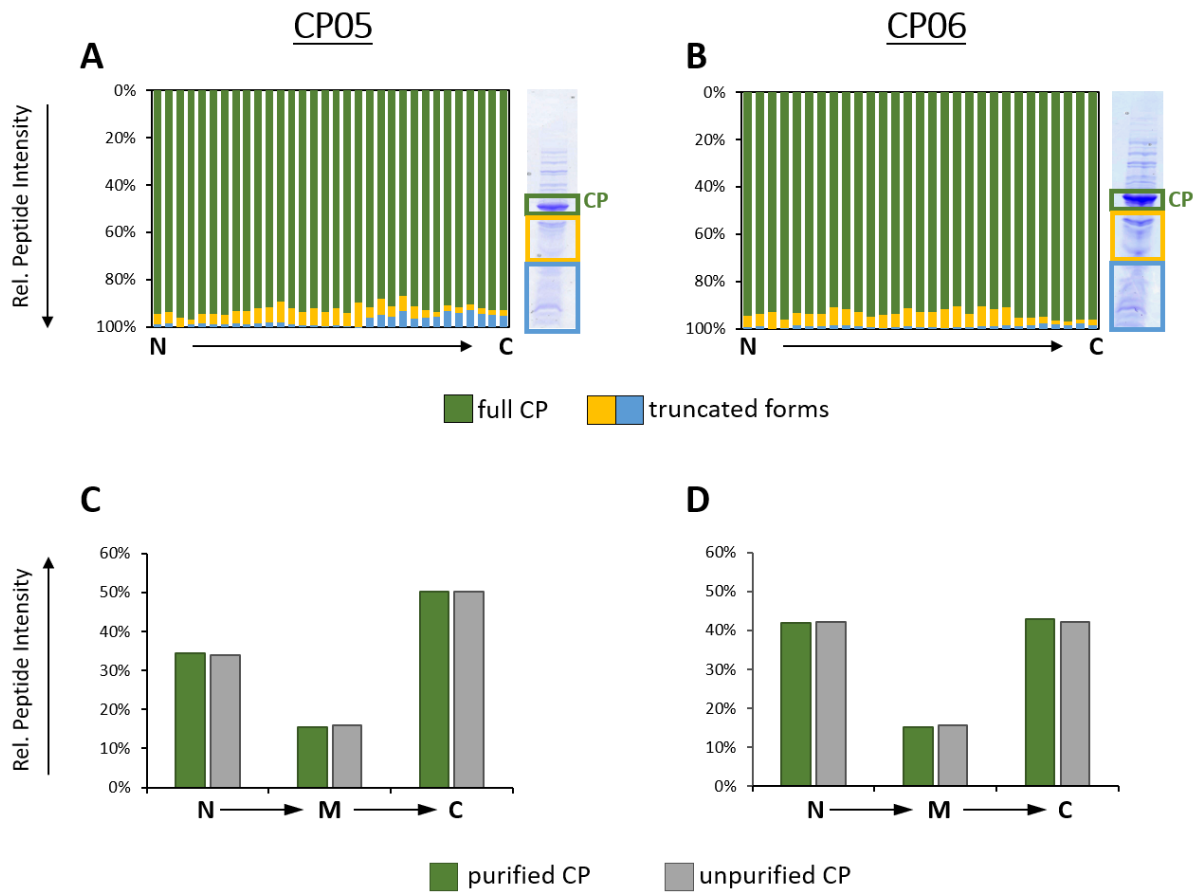

Figure S5. Truncation patterns for CP05 (A and C) and CP06 (B and D) standards.

A

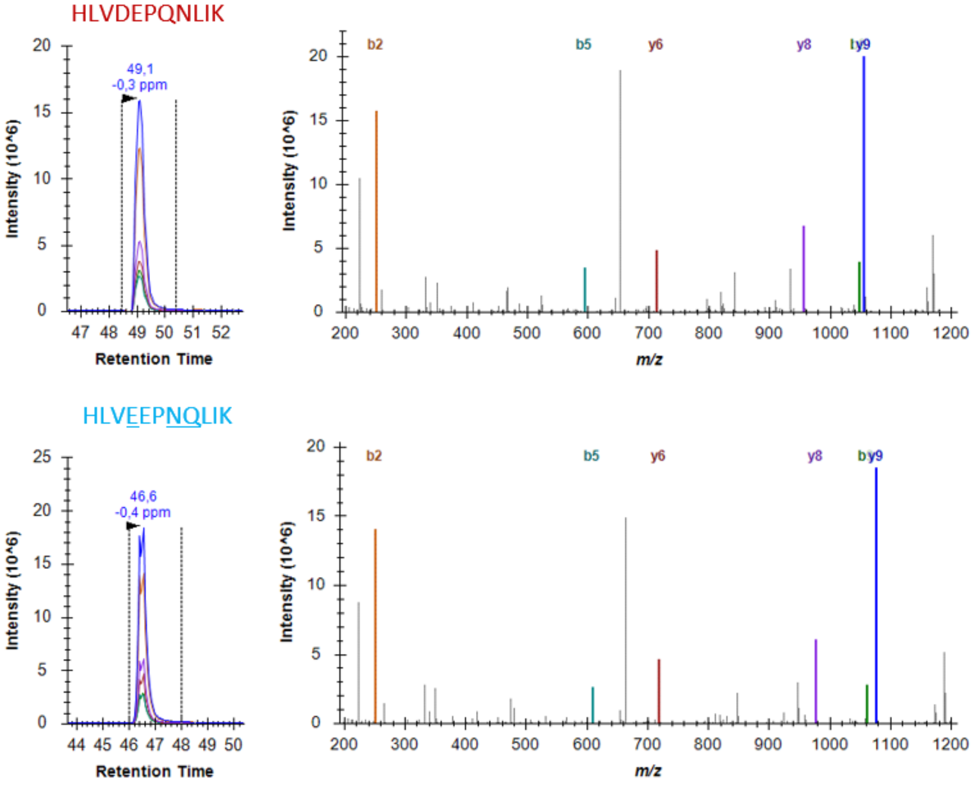
HLVDEPQNLIK
49,1
-0,3 ppm
Intensity (10^6)
Retention Time
b2
b5
y6
y8
y9
m/z
HLVEEPNQLIK
46,6
-0,4 ppm
Intensity (10^6)
Retention Time
b2
b5
y6
y8
y9
m/z

B

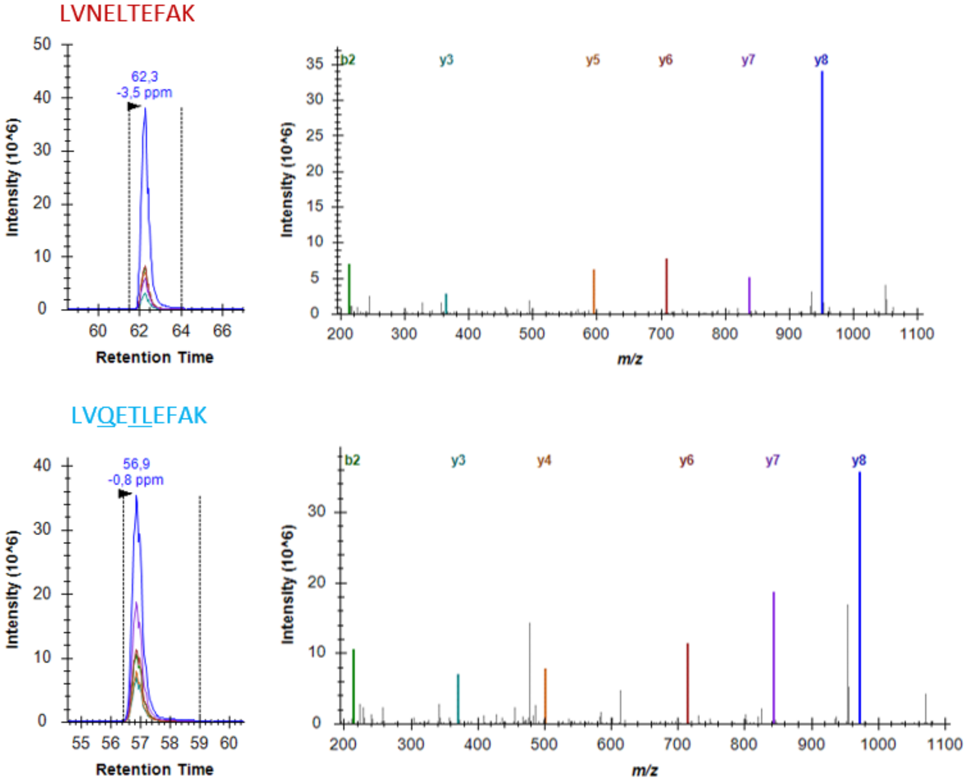
LVNELTEFAK
62,3
-3,5 ppm
Intensity (10^6)
Retention Time
b2
y3
y5
y6
y7
y8
m/z
LVQETLEFAK
56,9
-0,8 ppm
Intensity (10^6)
Retention Time
b2
y3
y4
y6
y7
y8
m/z

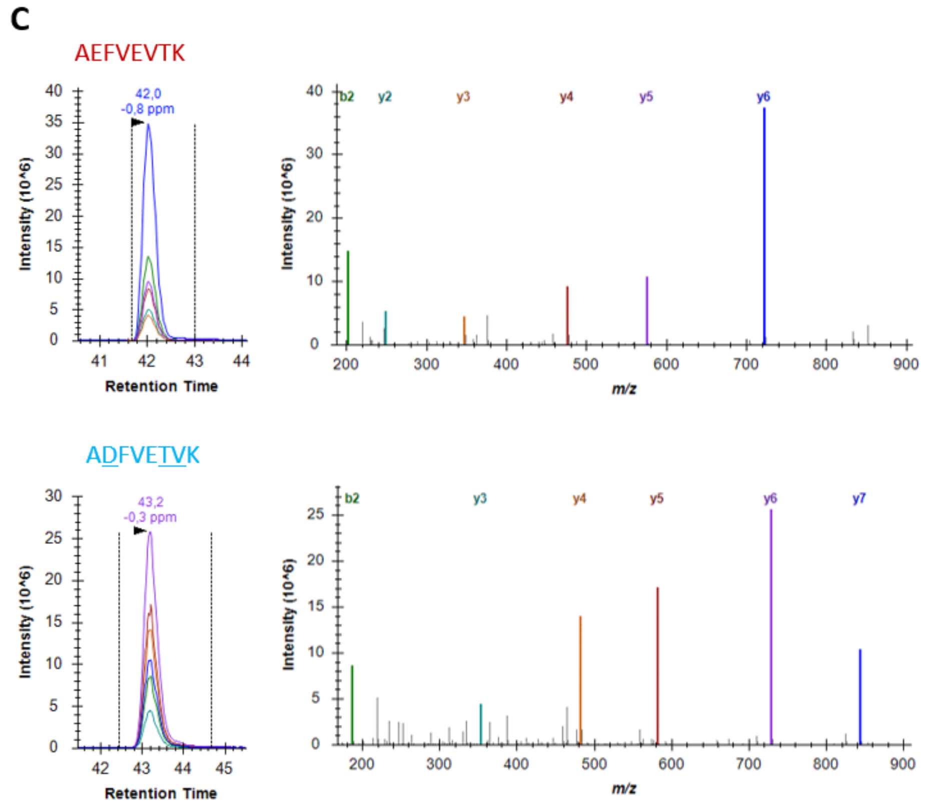
C
AEFVEVTK
42,0
-0,8 ppm
Intensity (10^6)
Retention Time
b2
y2
y3
y4
y5
y6
m/z
ADFVETVK
43,2
-0,3 ppm
Intensity (10^6)
Retention Time
b2
y3
y4
y5
y6
y7
m/z

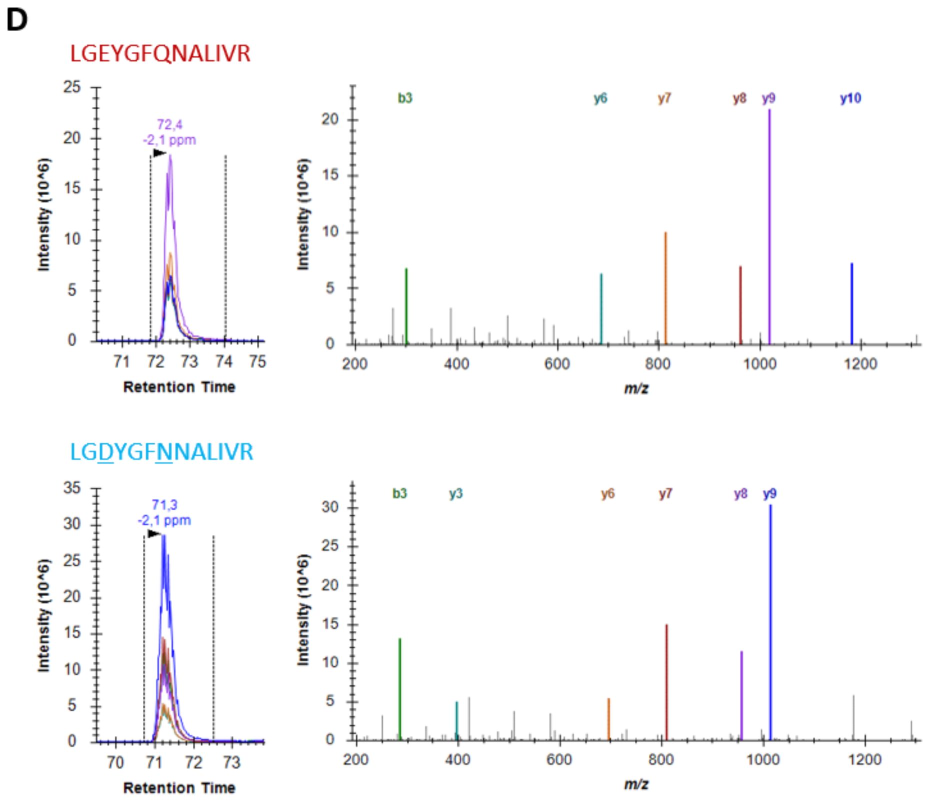
D
LGEYGFQNALIVR
72,4
-2,1 ppm
Intensity (10^6)
Retention Time
b3
y6
y7
y8
y9
y10
m/z
LGDYGFNNALIVR
71,3
-2,1 ppm
Intensity (10^6)
Retention Time
b3
y3
y6
y7
y8
y9
m/z

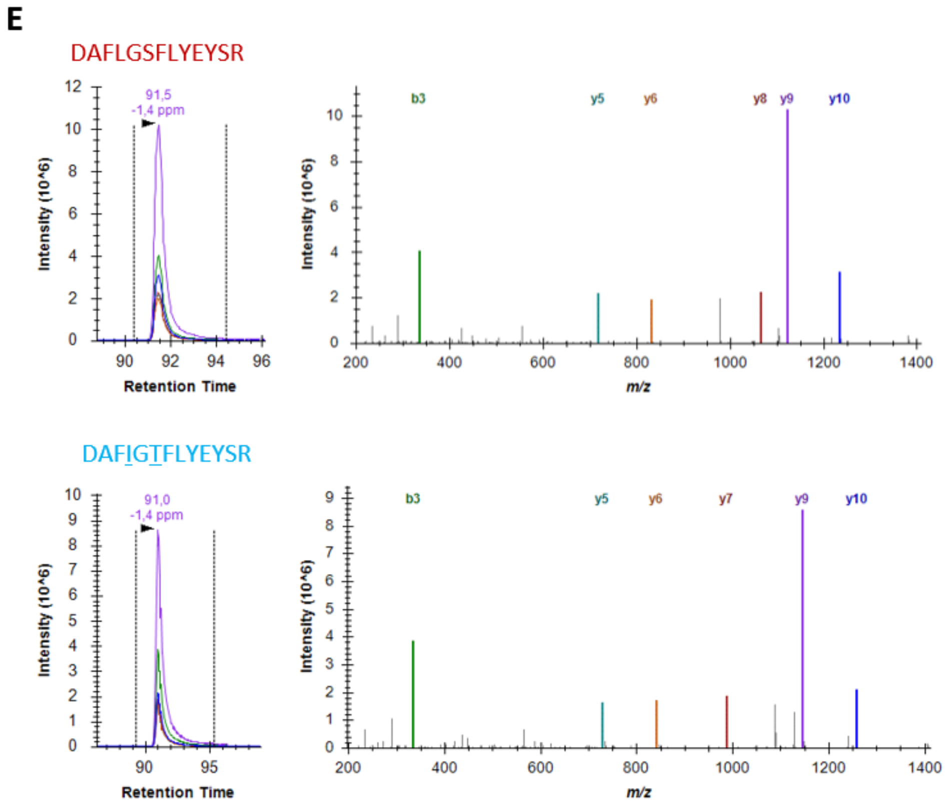

Figure S6. Tandem mass spectra of R-peptides in native (BSA, unlabeled) and scrambled forms (CP05/06, labeled) together with their chromatographic peaks: HLVDEPQNLIK (nat) and HLV<u>E</u>EP<u>NQ</u>LIK (scr) (**A**), LVNELTEFAK (nat) and LV<u>Q</u>E<u>TL</u>EFAK (scr) (**B**), AEFVEVTK (nat) and A<u>D</u>FVE<u>TV</u>K (scr) (**C**), LGEYGFQNALIVR (nat) and LG<u>D</u>YGF<u>N</u>NALIVR (scr) (**D**), DAFLGSFLYEYSR (nat) and DAF<u>I</u>G<u>T</u>FLYEYSR (scr) (**E**).

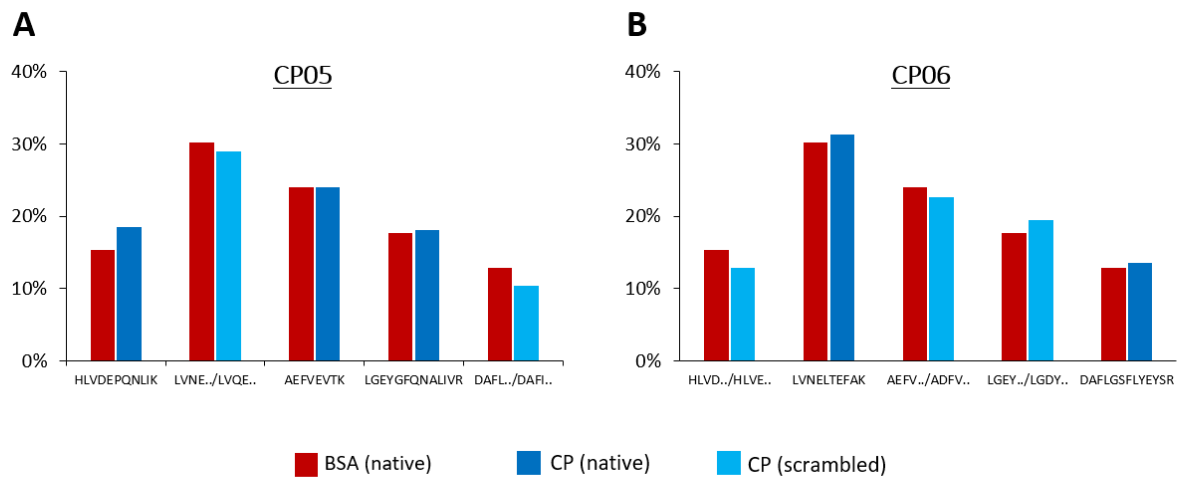

Figure S7. Matching relative peak areas of R-peptides for BSA (native) and both CP05 (**A**) and CP06 (**B**) obtained using Top6(y,b) fragment ions. Differences were <5 % for all pairs.

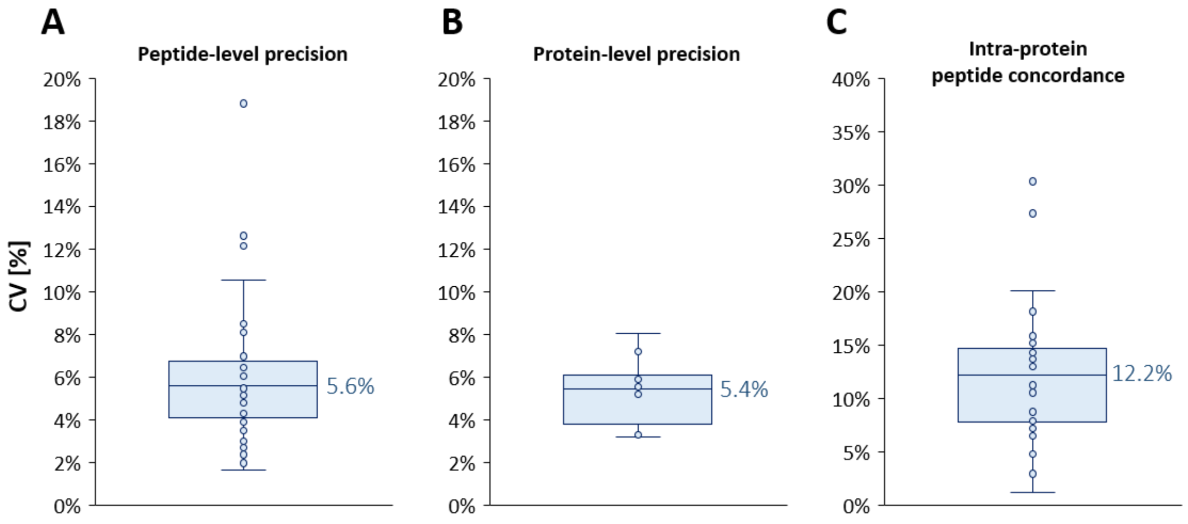

Figure S8. Precision of the FastCAT quantification estimated from the PRM analysis of the pooled CSF sample. Panels A and B show peptide-level and protein-level precision, respectively. The graphs are created with data acquired for all investigated proteins (10 target proteins and two CPs) based on three sample preparation replicates and two LC injections each. Panel C shows intra-protein peptide concordance obtained from the same data set. Median CV values are provided at the plots. Each box plot displays the median (line), the 25th and 75th percentiles (box), and the 5th and 95th percentiles (whiskers).

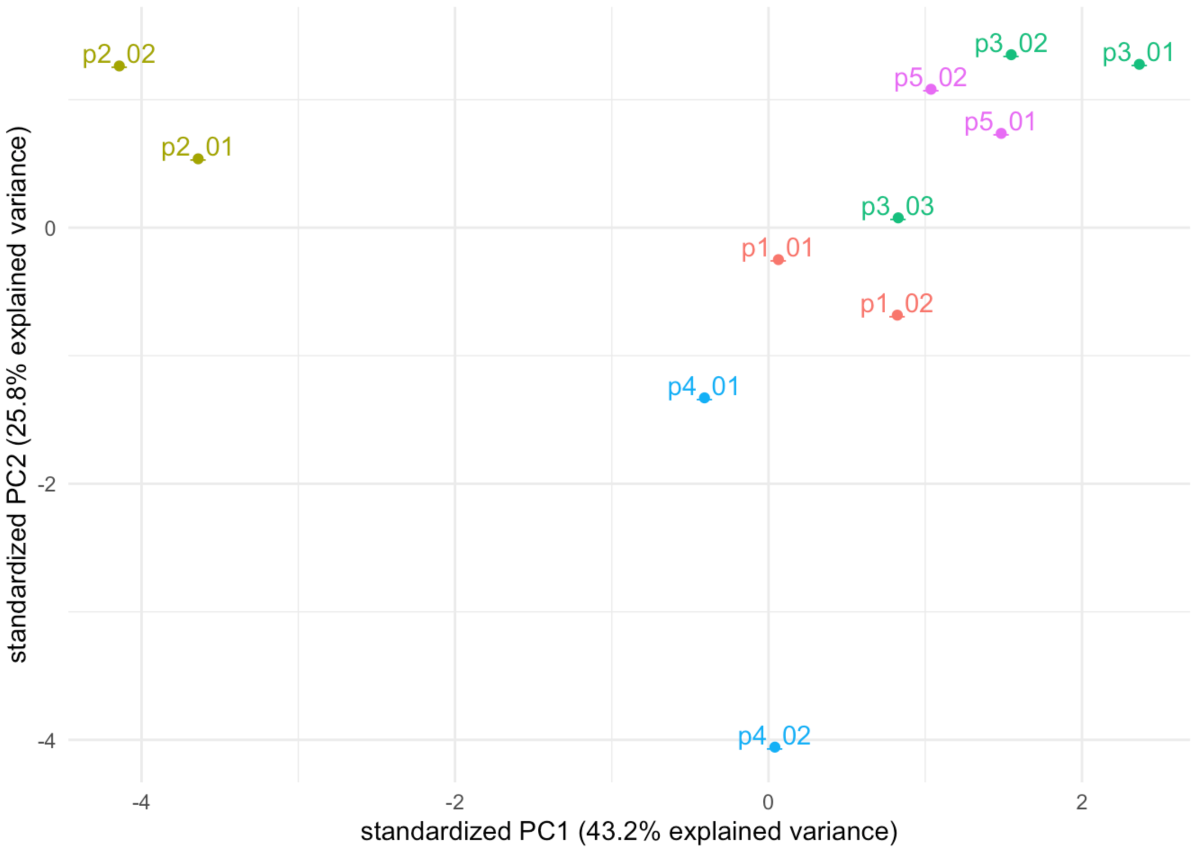

Figure S9. Principal component analysis (PCA) plot for all protein quantities (from Table S4) in patients 1-5 (p1-p5) with two (or three for p3) collections of samples. In this way, p2_01 and p2_02 stand for the first and second CSF collection from the patient p2, respectively.

Table S1. List of representative *E. coli* proteins (selected from most abundant proteins) used for estimating the amount of extra protein background introduced by spiking an unpurified CP standard, For each protein we provide UniProt accession number; sequences of peptides used for the quantification (three peptides per each protein); peptides retention times and charge states.

Protein	UniProt Accession	Peptide	RT	Charge state
DNA-directed RNA polymerase	P0A8V2	LIEVPVEYIAGK	76.7	2
		GSWLDFEFDPK	87.1	2
		LGDLPTSGQIR	57.5	2
Elongation factor Ts	P0A6P1	IGVLVAAK	53.2	2
		AEITASLVK	55.2	2
		EHNAEVTGFIR	53.7	3
50S ribosomal protein L4	P60723	DATGIDPVSLIAFDK	88.9	2
		SILSELVR	76.7	2
		DAQSALTVSETTFGR	69.9	2
Pyruvate dehydrogenase E1	P0AFG8	LVPIIADEAR	64.4	2
		AQYLIDQLLAEAR	89.5	2
		DWLQAIESVIR	105.7	2
50S ribosomal protein L1	P0A7L0	VGTVTPNVAEAVK	56.0	2
		QYDINEAIALLK	85.9	2
		GATVLPHGTGR	41.1	2
30S ribosomal protein S1	P0AG67	AYEDAETVTGVINGK	64.3	2
		GGFTVELNGIR	70.9	2
		SESAIPAEQFK	55.2	2
Elongation factor G	P0A6M8	IATDPFVGNLTFFR	89.9	2
		VYSGVVNSGDTVLNSVK	67.9	2
		GGVIPGEYIPAVDK	70.7	2
30S ribosomal protein S2	P0A7V0	VHIINLEK	51.9	2
		LENSLGGIK	51.7	2
		DAALSCDQFFVNHR	66.1	3
Elongation factor Tu1	P0CE47	TTLTAAITTVLAK	82.1	2
		AGENVGVLLR	61.4	2
		ILELAGFLDSYIPEPER	98.4	2

Table S2. Relative error of the CP05/06 quantification based on the scrambled R-peptides and using either MS1-derived (DDA) peptides peak areas or different MS2 fragment ions (PRM).

Native form	Scrambled form in CP	Δ RT [min]	CP with scrambled form	MS1-based (DDA)			MS2-based (PRM)						
				CP [fmol] by nat	CP [fmol] by scr	% error	CP [fmol] by nat	CP [fmol] by scr					
								Top1	% error	Top3(y)	% error	Top6(y,b)	% error
HLVDEPQNLIK	HLVEEPNQLIK	2.5	CP06	275	290	5.4%	294	241	17.9%	241	17.7%	236	19.7%
LVNELTEFAK	LVQETLEFAK	5.4	CP05	270	295	9.0%	280	180	35.8%	245	12.5%	250	10.9%
AEFVEVTK	ADFVETVK	1.2	CP06	275	274	0.4%	294	185	36.9%	268	8.7%	265	9.7%
LGEYGFQNALIVR	LGDYGFNNALIVR	1.1	CP06	275	312	13.4%	294	329	12.1%	341	16.4%	310	5.4%
DAFLGSFLYEYSR	DAFIGTFLYEYSR	0.5	CP05	270	188	31.0%	280	214	23.9%	203	27.4%	211	24.7%

Table S3. Protein concentrations (ng/mL) in 11 CSF samples from 5 patients diagnosed with multiple sclerosis. CSF was collected twice for four patients and three times for the patient #3. Patient age during the 1st collection is given in years (y), while the time difference (Δt) to the 2nd (and 3rd for patient 3) puncture is given in months (m).

Protein	Concentration in CSF [ng/mL]										
	**Patient 1**		**Patient 2**		**Patient 3**			**Patient 4**		**Patient 5**	
	time 1	time 2	time 1	time 2	time 1	time 2	time 3	time 1	time 2	time 1	time 2
	age, 43 y	Δt, 40 m	age, 61y	Δt, 17 m	age, 36	Δt, 10 m	Δt, 16 m	age, 30 y	Δt, 32 m	age, 30 y	Δt, 26 m
**APOE**	1489.28	1604.15	1106.19	922.07	1926.15	1807.81	1772.67	1854.96	2075.59	1996.34	1811.10
**APOD**	1197.68	1307.78	1185.15	1203.23	1284.59	1281.67	1297.31	1083.58	1060.41	1246.55	1327.29
**AACT**	759.84	684.61	765.58	723.96	580.30	564.96	770.17	810.35	1127.28	540.90	588.94
**ZAG**	100.05	113.18	102.01	96.32	85.98	85.89	87.58	102.12	108.05	85.19	85.76
**SCG1**	636.56	684.09	451.77	388.55	942.32	885.69	869.22	788.86	862.85	852.69	850.08
**LRG1**	67.98	71.42	77.14	80.84	50.02	47.78	114.29	38.45	92.91	27.13	33.82
**CH3L1**	51.77	35.09	35.16	33.37	53.82	49.85	44.63	38.90	27.48	27.90	25.74
**CNTN1**	238.90	256.07	159.70	148.28	229.68	230.81	229.77	241.20	268.62	257.04	246.15
**VGF**	139.34	163.29	62.64	60.65	175.94	133.49	141.99	73.35	98.14	110.97	99.89
**NPTX1**	75.67	86.02	42.50	37.18	77.01	72.52	71.98	64.28	81.85	70.87	68.53

Table S4. Chimeric protein (CP) standards used in this study together with their molecular weights, labeling efficiencies as well as amino acid sequences.

Chimeric Protein	Molecular weight	Labeling efficiency	Amino acid sequence
CP01	264.8 kDa	>99 %	MGSAWSHPQFEKGGGSGGGSGGSAWSHPQFEKLEVLFQGPAAAKVFADYEEYVKDFYELEPHKVAAAFPGD<br>VDRGLAGVENVTELKGSTLSAQQGSQFKNEPVSYILELINGRNIGDSYVAAKLDNGYNHLIEVVRLVLGIPTYG<br>RFSPLVASNERLTEAEGSSLYIGGRSTDAEEDPQVIKFADQDNDLVNLRLSNFVDTTVAWLNYRFFVSVTRVQL<br>TDLNRDVFIPDVFNNYKILAIFPFPGPSQYINVVPYLKVPIWPYTLYFRGTFFVSHKGHEISVFSLTHKAPLGLHF<br>HASWLKIINNPEATQRAVYWVEHVSRSHYHVGSALAKAEQNGYGVTVHYEELSSAKGSAYAHAENTLRSEGD<br>LTQITPPSALGVQVRAEVEAVQIIAESLKAANQLTDRPTIINVAISPSSDRGHGAGGADVLTYKDLNQGNSYVQD<br>KSLVFVDNHDNQRSEYTGLGAITEFRVVEFLDHLIDLGVAGFRIGALDTSRLIELLVRLADLLLQEERVNGEPVD<br>VGAFFIRITPDVVGAIVQEATTYFRATQLSEQLGSELTPKLLAADYADGVSQPRILSWNAVNLYGLKASEQPGL<br>TAIHTAFLRLLPAQYEDGISAPRGIATNDVGIAKDTFSFLTGSGRAINEGGFQSLPQKFGLNEGSEPQAYGIGLKI<br>TTHYTLNPRAILVYLVEKITPGWEENWAGALDVKINPQHSIPTLVDNGFTIWESRALGLEFNKINLDPYALYVIV<br>EKTNFDDSALFYASGESLKVLTTGGDAGSNRLGSDVQPPGRVFLQLLENQRNLSQTFGNIWRVFDLPDFLTWR<br>GSTVQFAARDGAISHVVFKYFNAYLSDRFSEPYDSTLSDVIKNVANLEATKDADAIVQQTLAKGTQAEIVVAD<br>VTKVGDVTEVAEAVAFLASSKSVLLTLRIDALEGVYKALNLDFDYKAITFPLFWENKLTLYGIDGSPPVRELPY<br>YEEANGSRAVGVELNKAPADPEAFKAIQVYLVEKLLNLQAGEHLKPEFLKINPQHTIPTLVDNGFALWESRGL<br>VVSGTRSLSDVSLTGRGNSYLILPPPRISGPSNHVTVVRNAAFSGDSYVSHRSVVNVGLGQALRSTQEEVDHIRS<br>AVHDVEVFLKVSTAIDAISVSGRVALQIQDVATSSRLEEISLRLLELHDNRLFNNFDVLRLAVLDLSHNRASLSG<br>IQSHAFKTFFDGNPIHTLRDFGVELEDLQITRSTISSTTTVRTSTTSSLTGNPRTSVATVAGGAVVGATKLLVPGS<br>SSTTTTSSLRLLPDTTDEQLLTSALEEKSTGNIFAAKVFAVNSAGRIFADNVYGRGVAEDFAPSFVKHDGGSPIT<br>GYIIEKHYPNPAVRVVGSEADTGRDAPTTESYLRSTVPFATSESNRLVGGNDLDTPLIITRIAELEGLGSRIQYLE<br>DSTRIGEAISQSSIRFITQIVDVTKAEDAGVYLVVARTYYDFGFVALDIIKVWQVSVSAHALLWDLNDGKDVLS<br>VAFSADNRLWDLAAGKLNNDLIARFQEALAGLSKLAAHDALGGAAKSAGSGVSTTAIEKIVQVQIDDVGKSEL<br>DVFSDWLQVARFSQSDFGLDQGETLLRESLLEITIYHQKGAAYQEAPVDAEVAVTPKISAFGLFTYSVFSIIAGS<br>LKVFANPVQLEFYGFVPWSIIQRQVGASSGSSGAVSKIVQIQQVGPAHLNVKVQDNSVLVEGNHEERVLPAIEA<br>FQKFSEATLDEIIRFHGVALAFNALDSKENAILTDIWNITPFKVIVDILLKTILVDLQVGKLAELHAASVVAKEAG<br>LEIELAPKAGFAGDDAPRSYELPDGQVITIGNERVAPEEHPVLLTEAPLNPKGYSFTTTAEREITSLAPSTIKEITA<br>LAPSTIKQEYDESGPGIVHRQEYDESGPSIVHRSIELLEKLQASGDFPTTKEQNSPIYISRATVAAFAASEGHAHPR<br>YQVIVKSSDAQSQATASEAESKALSASSLIALSSRDPVLTAFQLSWELKDDYGITEDDIIEVRATAWVIVHRVEL<br>ELWLKVVLPDLAVLRALGIEEQSGGAARVDEYGFFLYWKYYLSDLARIEQADYLPTEQDILRVPTTGILEYPFD<br>LDGIVFRTIITYPWFQNSSVILFLNKAGIAVEGDIKDAFGIIVSYAVKDTALASTTLIASQDARDFLLSPGELELEV<br>TLDKVFGQLATTYRLQYAPLNRISLEVTLDRLLQPAPGTIEKFYPEDVAEELIQDITLRIGFPWSEIRTTHTAGFL<br>ANDRLFYLQVKAAIELNRGDNILLTKVPPEVPPSKLVDFIINRSLVGVQKQIDVETVADRYIQATDLSEASRNAE<br>YDQFLEDIQKATAWVIVHRALSASSLIALSSRHLVDEPQNLIKYLYEIARQTALVELLKLGEYGFQNALIVRDAF<br>LGSFLYEYSRLVNELTEFAKGSGHHHHHH

CP02	79.3 kDa	>99 %	MGSAWSHPQFEKGGGSGGGSGGSAWSHPQFEKLEVLFQGPAAAKVFADYEEYVKDFYELEPHKVAAAFPGD<br>VDRGLAGVENVTELKLGDYGFNNALIVRDAFIGTFLYEYSRAAAYVLQETPVVNALVDENEIVYRSDGEVEIA<br>SEKVIDTAYEIIKGIILIGEGIGNAEEQAAEFLKVQNDDSIVFFDYRVLEATLAQDFSKLVTWYDNEFGYTNRYA<br>GEDAAAGAAETLFVAKAEIEAVQIIAETLKVVEFLEHILDLGVAGFRIVQVNLDDVGKVGDVTEVAEAAVFLA<br>TSKAGLAIEGDIKANEILSDIWNITPFKGIAEDFAPTFVKIADLEGIYKALQLDFEYKALVFETWQGPLEVRWVAI<br>DGEQYGEGSSRLIDDNVANALKVVDHAYEAVVIGAGGAGLRYLYDVARFHGATSINLVGDLDTVTNPKGTVA<br>HDGDYLIVAKTVEADAAHGSVTRTIEADAAHGSVTRVVELITYIATKYAVFDTGSRVTENVLAFIYKQLLFSAG<br>AELNKLDLGTVVSPVSGPKLGANTLLELVIFGRAFGGNTQDFGRVFQFLEASAGSKLNADTSLFILASKGQETST<br>QPIATIFAWSRAGQSHLGLPIFGSAVEAKGVEPSHAISGARALIANGTGPYFYLPKNTVIASGGYGRAAAAQINY<br>IRSGNVVPGYHGAVLRVALLGAGAGIGNPLGLLLKVPQVILAVGLPARHLVEEPNQLIKLVNELTEFAKGSGHH<br>HHHH
CP03	42.3 kDa	>99 %	MGSAWSHPQFEKGGGSGGGSGGSAWSHPQFEKLEVLFQGPAAAKVFADYEEYVKDFYELEPHKVAAAFPGD<br>VDRGLAGVENVTELKIGEEYISDLDQLRVIFLENYRLLSYVDDEAFIRVLVDLERTLSDYNIQKESTLHLVLRTIT<br>LEVEPSDTIENVKEGIPPDQQRIGDYAGIKVLGIDGGEGKEALDFFARVVGLSTLPEIYEKANELLINVKGVIFYE<br>SHGKGNPTVEVELTTEKTFAEALRAADALLLKTAGIQIVADDLTVTNPKNVNDVIAPAFVKVNQIGTLSESIKA<br>VDDFLISLDGTANKHLVDEPQNLIKYLYEIARQTALVELLKLGEYGFQNALIVRDAFLGSFLYEYSRLVNELTEF<br>AKGSGHHHHHH
CP04	43.0 kDa	>99 %	MGSAWSHPQFEKGGGSGGGSGGSAWSHPQFEKLEVLFQGPAAAKVFADYEEYVKDFYELEPHKVAAAFPGD<br>VDRGLAGVENVTELKHLVDEPQNLIKLVNELTEFAKIGEEYISDLDQLRVIFLENYRLLSYVDDEAFIRVLVDLE<br>RTLSDYNIQKESTLHLVLRTITLEVEPSDTIENVKEGIPPDQQRYLYEIARLVTDLTKAEFVEVTKIGDYAGIKVL<br>GIDGGEGKEALDFFARVVGLSTLPEIYEKANELLINVKGVIFYESHGKGNPTVEVELTTEKTFAEALRAADALLL<br>KTAGIQIVADDLTVTNPKNVNDVIAPAFVKVNQIGTLSESIKAVDDFLISLDGTANKLGEYGFQNALIVRDAFLG<br>SFLYEYSRGSGHHHHHH
CP05	45.4 kDa	>99 %	MGSAWSHPQFEKGGGSGGGSGGSAWSHPQFEKLEVLFQGPAAAKVFADYEEYVKDFYELEPHKVAAAFPGD<br>VDRGLAGVENVTELKHLVDEPQNLIKLVQETLEFAKSELEEQLTPVAEETRLGPLVEQGRAATVGSLAGQPLQ<br>ERQWAGLVEKLAVYQAGARNILTSNNIDVKIPTTFENGRNPNLPPETVDSLKVLNQELRWYEIEKAEFVEVTKI<br>TLLSALVETRNLAVSQVVHKAVLDVFEEGTEASAATAVKADLSGITGARHVEDVPAFQALGSLNDLQFFRYSL<br>TYIYTGLSKEIPAWVPFDPAAQITKQKWEAEPVYVQRNYPSLELDKNYLNYGEEGAPGKGEAGAPGEEDIQGP<br>TKWQQQGDLQDTKLGEYGFQNALIVRDAFIGTFLYEYSRGSGHHHHHH
CP06	46.0 kDa	>99 %	MGSAWSHPQFEKGGGSGGGSGGSAWSHPQFEKLEVLFQGPAAAKVFADYEEYVKDFYELEPHKVAAAFPGD<br>VDRGLAGVENVTELKHLVEEPNQLIKLVNELTEFAKVAAGAFQGLRGQTLLAVAKTLDLGENQLETLPPDLLR<br>ENQLEVLEVSWLHGLKFPLTNAIKILGQQVPYATKTLLSVGGWNFGSQRVTIDSSYDIAKGNQWVGYDDQESV<br>KADFVETVKFIPLIPIPERASPFPVYKTDGAAPNVAPSDVGGGGGRVQVTSQEYSARNSEPQDEGELFQGVDPRT<br>HLGEALAPLSKLADLASDLLLQYLLQGGARVGEEDEEAAEAEAEAEEAERFQLTFPLRTPAAETLSQLGQTLQS<br>LKALSGNVIAWAESHIEIYGGATKLGDYGFNNALIVRDAFLGSFLYEYSRGSGHHHHHH
